# Mountains of the Mist: A first plant checklist for the Bvumba Mountains, Manica Highlands (Zimbabwe-Mozambique)

**DOI:** 10.3897/phytokeys.145.49257

**Published:** 2020-04-10

**Authors:** Jonathan Timberlake, Petra Ballings, João de Deus Vidal Jr, Bart Wursten, Mark Hyde, Anthony Mapaura, Susan Childes, Meg Coates Palgrave, Vincent Ralph Clark

**Affiliations:** 1 Biodiversity Foundation for Africa, 30 Warren Lane, East Dean, E. Sussex, BN20 0EW, UK Biodiversity Foundation for Africa East Dean United Kingdom; 2 Flora of Zimbabwe & Flora of Mozambique projects, 29 Harry Pichanick Drive, Alexandra Park, Harare, Zimbabwe Flora of Zimbabwe & Flora of Mozambique Harare Zimbabwe; 3 Meise Botanic Garden, Bouchout Domain, Nieuwelaan 38, 1860, Meise, Belgium Meise Botanic Garden Meise Belgium; 4 Afromontane Research Unit & Department of Geography, University of the Free State, Phuthaditjhaba, South Africa University of the Free State Phuthaditjhaba South Africa; 5 National Herbarium of Zimbabwe, Box A889, Avondale, Harare, Zimbabwe National Herbarium of Zimbabwe Harare Zimbabwe; 6 Box BW53 Borrowdale, Harare, Zimbabwe Unaffiliated Harare Zimbabwe

**Keywords:** endemics, floristics, invasive species, Manica Highlands, montane, plant diversity

## Abstract

The first comprehensive plant checklist for the Bvumba massif, situated in the Manica Highlands along the Zimbabwe-Mozambique border, is presented. Although covering only 276 km^2^, the flora is rich with 1250 taxa (1127 native taxa and 123 naturalised introductions). There is a high proportion of Orchidaceae and Pteridophyta, with both groups showing a higher richness than for adjacent montane areas, which may be due to the massif’s relatively high moisture levels as a result of frequent cloud cover. However, in contrast to other mesic montane regions in southern Africa, there are relatively few near-endemic or range-restricted taxa: there is only one local endemic, *Aeranthes africana*, an epiphytic forest orchid. This is likely to be an effect of the massif having limited natural grassland compared to forest, the former being the most endemic-rich habitat in southern African mountains outside of the Fynbos Biome. Six other near-endemic taxa with limited distribution in this portion of the Manica Highlands are highlighted. The high number of invasive species is probably a result of diverse human activities in the area. The main species of concern are *Acacia melanoxylon*, a tree that is invading grassland and previously cultivated land, the forest herb *Hedychium gardnerianum* which in places is transforming forest understorey with an adverse effect on some forest birds, and the woody herb *Vernonanthura polyanthes* which invades cleared forest areas after fire. Future botanical work in the massif should focus on a more detailed exploration of the poorly known Serra Vumba on the Mozambican side and on the drier western slopes. This will allow for a more detailed analysis of patterns of endemism across the Manica Highlands.

## Introduction

Southern African mountains continue to fascinate biologists, ecologists and conservationists with their high endemism, high species diversity, and as a haven for taxonomically complex and cryptic evolutionary lineages (White 1978; Taylor et al. 2013; Uys and Cron 2013; Conradie 2014; Mynhardt et al. 2015; Padayachee and Procheş 2016; Phiri and Daniels 2016; Conradie et al. 2018; Branch et al. 2019). From a floristic perspective, there has been a steady output of comprehensive data from the region over the past 25 years, for example the Nyika Plateau (Burrows and Willis 2005) and Mount Mulanje (Strugnell 2006) in Malawi; Mounts Gorongosa, Mabu and Namuli (Müller et al. 2008; Timberlake et al. 2009; Bayliss et al. 2014; Timberlake, in prep.) in Mozambique; Chirinda Forest (Drummond and Mapaure 1994) in Zimbabwe; the Angolan Highlands (Goyder and Gonçalves 2019); the heterogeneous southern African Great Escarpment (Clark et al. 2011, 2014; Roth et al. 2014; Darbyshire et al. 2018; Carbutt 2019). This has greatly improved our regional understanding of montane floristics, patterns of endemism, biogeography and conservation needs. In addition, an account of all the endemic and near-endemic plants from Mozambique has recently been published (Darbyshire et al. 2019b), some of which occur in these border areas. Despite these advances, ongoing biodiversity research in southern African mountains remains a key regional need (Clark et al. 2011, 2019; CEPF 2012), and there remains a substantial lag in the production of fundamental biodiversity and taxonomic data compared to other mountains in Africa.

The Manica Highlands (Clark et al. 2017), which lie on the border between Zimbabwe and Mozambique and are mostly synonymous with Van Wyk and Smith’s (2001) Chimanimani-Nyanga Centre of Floristic Endemism, comprise an area that has been well-botanised over the last 100 years, yet with few publications. Over the past decade, attention has been focused on improving our knowledge of plant diversity and endemism for this ecologically complex 8,000 km^2^ mountain system. For instance, the first comprehensive floristic treatment of the Nyanga massif was published only recently (Clark et al. 2017), as was the first substantial revision in 50 years of the Chimanimani flora (Wursten et al. 2017).

The central parts of the Manica Highlands (from north to south: Stapleford, Penhalonga, Bvumba, Banti, Himalaya, Tsetserra) are now the outstanding areas that require synthesis of available data and further fieldwork, with the Bvumba being probably the most thoroughly botanised component of the Manica Highlands. Here we present the first comprehensive plant checklist for the Bvumba massif, with some notes on the massif and its flora.

## The Bvumba area

### Defining the study area

Clark et al. (2017) defined the Bvumba as the entire central component of the Manica Highlands, which includes the Bvumba as well as the Penhalonga and Stapleford uplands that occur immediately to the north, i.e. between the Bvumba and Nyanga. However, due to lack of adequate floristic data for Penhalonga and Stapleford, we here restrict ourselves to the Bvumba massif *sensu stricto* and not to the broader Bvumba area as shown in Clark et al. (2017). The checklist area is defined as that part lying primarily above 1200 m altitude with significant vegetation cover, an extent of around 276 km^2^, and differs slightly from the more rigid use of the 1200 m contour used by Childes and Mundy (2001).

### Location and topography

**Figure 1.**
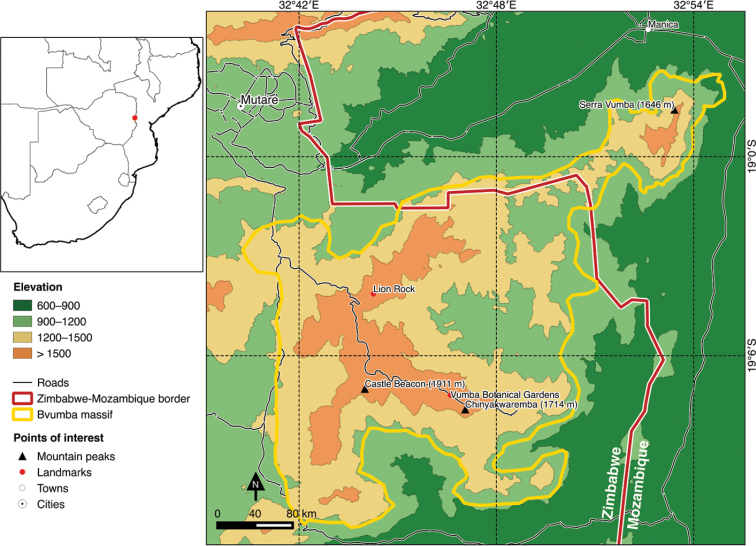
Map of the geographic location and selected elevations of the Bvumba massif and vicinities, with main points of interest.

Centred on 19°06'S, 32°47'E, the Bvumba lies 20 km south-east of the border city of Mutare and straddles the Zimbabwe-Mozambique border (Figure 1). The largest extension lies within Mutare District in eastern Zimbabwe, but a significant area of around 30 km^2^ lies over the border in Manica District in Mozambique’s Manica Province. This north-eastern extension terminates at the peak of Serra Vumba (1646 m) at 18°58'35"S, 32°53'25"E, just 5 km south of Manica town (previously Vila da Manica) on the main Mutare‒Beira highway. The area as a whole is bounded in the north by the Muneni valley (in which the Forbes/Machipanda border post is situated) and in the south by the Burma valley (Nyamataka River), which separates it from the Banti-Himalaya-Tsetserra massif. The Mozambique midlands/lowlands and Chicamba Real dam form the eastern limits, while the Odzi River valley forms the western boundary. Outlying ridges and inselbergs to the west − including Cecil Kop – have been excluded. The lower elevational cut-off of approximately 1000–1200 m used here roughly follows the base of the Bvumba massif where it emerges from the surrounding plains.

Consisting of an upland massif, the highest points in the study area are Castle Beacon (1911 m) and Chinyakwaremba (1714 m), while the main rivers are the Nyamataka in the south, which drains into the Rio Vanduzi via the Chicamba Real dam and then on into the Rio Búzi, the Zonwe River in the centre and the Ndonwe River in the north, both also draining into the Vanduzi. To the west the main river is the Nyachowa, which drains into the Odzi and eventually into the Save River.

### Geology

The principal rocks underlying the Bvumba area are gneissic granites of Proterozoic age, perhaps 2560 million years old, interlaced with intrusions of finer-grained darker dolerite rock perhaps 1800 million years old (Bartholomew 1990; Martin 2000). Millions of years of erosion have left the granite domes exposed, standing above the surrounding younger landscape. Some peri-glacial features date from the Pleistocene period (25,000 years BP). No minerals of economic significance are noted, although gold has been mined for hundreds of years from areas just north of Mutare. The soils are often deep and well-weathered, but are not considered particularly fertile owing to their age and weathering.

### Climate

Surprisingly, there does not appear to be a long-term rainfall recording station in the Bvumba area (Agritex 1989), the nearest being at Mutare, which is significantly lower in elevation and with a lower annual rainfall. On isohyet maps, rainfall is indicated as being around 1800 mm/year (Agritex 1989). The warm rainy or growing season extends from November to mid-March, with a colder dry season from June through to August. Frosts are scarce. A major feature, and one which gives the mountains their name of Mountains of the Mist, are the frequent mists and low moist cloud during the dry season, sometimes called *guti* (Martin 2000). It is these mists that reduce the physiological stress of the dry season for many plant species and allow forest to thrive.

### History and land use

The area has been settled, on the Mozambique side in particular, for perhaps 1000 years, with the first written record of people living there dating from Portuguese explorers in the first half of the 17^th^ century (Bannerman 2010; Martin 2000). It was possibly more heavily settled in the past than it is now, as seen from the many grinding stones found inside the forests. When European occupation first started, much of the area was settled by people of the Chirara dynasty (Martin 2000), particularly on the Mozambique side and along the Nzombe valley. The first European settlement on the Zimbabwe side started around 1890, and the first formal concession was granted by the British South African Company for the farm Cloudlands in 1898, now a private nature reserve. A road was cut in 1917 to the Bunga Forest from Mutare (then Umtali), and by 1921 the road continued over and into the Burma Valley (Martin 2000).

Within Zimbabwe, the Bvumba area is now nearly all on privately held land, much of it in small agricultural holdings, plantations or managed for conservation or ecotourism, including the famous Leopard Rock Hotel and golf course. There is a good road network and the area is well-settled with many properties, a number of which are used as holiday homes with many owners making strong efforts to conserve both the flora and bird life. Commercial farming in the Bvumba area includes dairy, *Protea* flowers, coffee, and with some wattle and *Eucalyptus* plantations. The only formal conservation areas are the Bvumba National Park and Botanic Gardens (201 ha) and the now much-expanded Bunga Forest Botanical Reserve (1,560 ha) managed by the Zimbabwe Parks and Wildlife Management Authority. There are no formally conserved areas on the Mozambique side, although from recent Google Earth imagery much of Serra Vumba’s vegetation cover appears to be relatively intact and it may be protected by local traditions.

### Vegetation

There are four broad vegetation types occurring in the Bvumba area ‒ montane grassland, Afromontane evergreen forest, high-rainfall miombo woodland, and secondary scrub savanna (Martin 2000). The edges of the forests are surrounded by a dense scrub of *Pteridium*, *Smilax*, *Buddleja*, *Vangueria* and *Vernonia* (Childes and Mundy 2001). In addition, there are plantations of exotic timber trees and cultivated or fallow agricultural land. It is possible that montane forest originally covered much of the Bvumba, principally owing to dry season precipitation in the form of low wet clouds (*guti*). But there is little montane grassland, a vegetation type of great botanical significance in the Nyanga and Chimanimani areas (Timberlake et al. 2016a). It is not clear why this is so, but it might be due to the somewhat lower elevations of the Bvumba compared to Nyanga and/or the greater amount of winter precipitation that allows forest to sustain itself. The high-rainfall woodlands contain many epiphytic ferns and orchids, which also have a high frequency in montane forest. Burrows (1990) suggests that the Bvumba has perhaps the richest fern flora in southern Africa owing to the pervasive mists. Secondary scrub savanna is generally found on infertile or gravelly soils or in degraded forests or reverted agricultural land, and it is here that a number of invasive tree and shrub species are found (S. Childes, pers. obs.).

### Previous studies

The Bvumba has seen much botanical activity and many collections over the last 50 years, but almost entirely from the Zimbabwe side. Two notable collectors were Norman Chase, who assembled over 8,000 specimens with meticulous notes, mostly from the Manica Highlands but with a large number from the Bvumba, and John Ball, for whom the Bvumba and Chimanimani provided the inspiration for his book on epiphytic orchids (Ball 1978). Other significant collectors include Hiram Wild, Darrel Plowes, Tom Müller and Hamish Gilliland. Three of the current authors (Mark Hyde, Petra Ballings and Bart Wursten) have collected extensively there over the last 20 years, and another (Susan Childes) runs a small forest and bird conservation area. Although no comprehensive overall checklist was available, detailed lists of the orchids and ferns of the broader area are found on the Vumba Nature website (Ballings and Wursten 2019) and a preliminary Bvumba plant list was compiled by Mark Hyde from a manuscript list by Norman Chase (Chase, no date) and Tree Society and Orchid Society records (Hyde 1999).

The Bvumba Highlands are considered to be an Important Bird Area (IBA ZW004, Childes and Mundy 2001), known for the richness of its montane avifauna. The IBA is taken to be the area above 1200 m elevation, considered to be the lower limit for montane bird species (Harwin et al. 1994). A total of 242 bird species have been recorded, including three of global conservation concern.

The only detailed plant ecological work done so far specifically in the Bvumba area is that by Plowes (2002), who looked at the impacts of the devastating Cyclone Eline on the 40-ha Bunga Forest in February 2000. He noted nearly 200 fallen trees that had created 46 patches totalling 1.57 ha, equivalent to a loss of 13% of forest cover. Gilliland (1938), in his study of the vegetation of Zimbabwe’s Manicaland, surprisingly does not mention areas this far south.

During his major study on Eastern Zimbabwe’s moist forests (Müller 1999, 2006), Tom Müller recorded 37 georeferenced 50 × 50 m forest plots in the Bvumba area. These fell into seven of his 12 described forest types, with most being Type 5 (Syzygium guineense subsp. afromontanum montane forest, 10 plots), Type 7 (Mixed sub-montane forest, 8 plots) and Type 11 (Medium elevation forest, 8 plots). In addition, there were a few plots each of Type 6 (Regenerating montane forest), Type 8 (*Craibia brevicaudata* forest), Type 9 (*Albizia*-dominated regenerating forest) and Type 10 (*Albizia schimperiana* forest). Hence most of the Bvumba forest plots recorded fell into what Muller calls sub-montane forest, with only a few from the montane or medium elevation forest zones. An interesting finding is that of the 37 plots marked on aerial photographs from the early 1970s, only three have been obviously lost or damaged from what can be seen of their canopy cover using recent Google Earth imagery (most dating from June 2019).

Although not formally documented, it does appear that disturbance over the last 100 years has led to a drying out of some of the forests and an invasion of alien plants (T. Müller, pers. comm. 2017). The impression (S. Childes, pers. obs.) is that the fern flora is moving towards the more generalist species and that some drought-sensitive species of angiosperms such as *Streptocarpus umtaliensis* and *Cryptostephanus vansonii* are reducing.

## Materials and methods

As the Bvumba is well-documented botanically, with approximately a century of botanical collecting, a checklist approach to documenting plant diversity and endemism was decided upon in order to be comparable to floristic lists recently compiled for Nyanga massif (Clark et al. 2017) and the Chimanimani mountains (Wursten et al. 2017). The two main sources were (i) an extract of the Harare Herbarium (SRGH) database of all records containing the word ‘Vumba’ in the locality field, with any records obviously from below 1200 m elevation removed, and (ii) records from the Bvumba area above 1200 m elevation cited on the Flora of Zimbabwe website (https://www.zimbabweflora.co.zw/, Hyde et al. 2020). In addition to these there were (iii) records from published volumes of Flora Zambesiaca, (iv) orchid and pteridophyte records listed on the Nature Vumba website (Ballings and Wursten 2019), (v) confirmed records from an unpublished Bvumba checklist (Hyde 1999) including those cited as being from Chase’s list (Chase, no date), (vi) any additional records from forest plot studies undertaken by Müller in the 1970s (Müller 2006), and finally (vii) personal records from the authors. A herbarium specimen or record citation (i.e. from the Flora of Zimbabwe website) is given for each taxon, or a confirmed sighting (s.r.) indicated. If there was any uncertainty over an occurrence, the record was omitted.

Families and species are listed alphabetically under pteridophytes, gymnosperms, monocotyledons and dicotyledons. Nomenclature and family arrangement follow those used on the Flora of Zimbabwe website (Hyde et al. 2020, accessed 1 February 2020). Pteridophyte families follow that used in the Pteridophyte Phylogeny Group (2016). Species authorities are abbreviated following Brummitt and Powell (1992). Synonyms are given only for significant recent changes or for taxa that have been known or recorded locally under a different name (e.g. in Mapaura and Timberlake 2004) or where confusion may occur. Where a taxon is believed to be endemic or near-endemic, this is indicated with an E or NE, respectively. Species that are said to be naturalised or introduced on the Flora of Zimbabwe website are indicated with an asterisk (*).

## Results and discussion

The checklist contains 1250 taxa, comprising 137 pteridophytes, 2 gymnosperms and 1111 flowering plants (Table 1). Of these, 1127 are native species and 123 (9.8%) are naturalised or semi-naturalised introductions, most, unsurprisingly, being cosmopolitan weeds in the Asteraceae (25 species), Poaceae (8 species) and Amaranthaceae (7 species). The largest families represented in the checklist are shown in Table 2.

In terms of species, there are three findings of particular note. First, there is a particularly high number of orchids (125 taxa across 276 km^2^), higher than might have been expected and significantly more than the number found in the more extensive Nyanga area above 1200 m elevation (92 taxa across 2181 km^2^; Clark et al. 2017) and in the Chimanimani mountains (97 taxa across 530 km^2^; Wursten et al. 2017). There are also a large number − 137 taxa − of pteridophytes (particularly ferns), compared to 136 taxa in the Nyanga area and just 105 in the Chimanimani. This is probably due to the greater moisture levels and frequent clouds in the Bvumba; the area is said to be possibly the richest locality for pteridophytes in southern Africa (Burrows 1990).

The third point of interest, again possibly linked to the high precipitation levels, is the lack of the montane conifer *Widdringtonia nodiflora* on the Bvumba, a species that is locally common both in the Nyanga and Chimanimani areas as well as on Mt Mulanje in southern Malawi (where it also occurs with *W. whytei*) and Mt Gorongosa in Mozambique (Müller et al. 2008). *Widdringtonia* is generally found on the drier rain-shadow side of these large mountains.

**Table 1.** Total number of taxa and introduced taxa in the Bvumba checklist, by group.

	No. taxa	No. introduced
Pteridophytes	137	1
Gymnosperms	2	1
Monocotyledons	336	15
Dicotyledons	775	106
TOTAL	1250	123

**Table 2.** The 10 largest families represented on the Bvumba checklist.

**Family**	**No. taxa**
Orchidaceae	125
Asteraceae	119
Fabaceae *sensu lato*	93
Poaceae	93
Rubiaceae	59
Acanthaceae	33
Lamiaceae	29
Cyperaceae	28
Aspleniacaeae (Pteridophyta)	27
Apocynaceae	22

### Endemics and taxa of restricted distribution

There is only one taxon known to be endemic to the Bvumba, the epiphytic orchid *Aeranthes africana*. Noted just twice, it is apparently now not found in its first-recorded location in the forests near Castle Beacon (S. Childes, pers. comm.). This species was recently assessed using IUCN Red List criteria (IUCN 2001) as Critically Endangered (Timberlake 2020, in press) and could be almost extinct, but it is cryptic among the leaves of *Podocarpus milanjianus* and difficult to see except on fallen branches so may have been overlooked.

Six other near-endemic taxa – here defined as taxa found only on nearby montane massifs such as Nyanga, Serra Choa, Stapleford, Banti/Himalaya and Tsetserra, but not including those also found on the Chimanimani or further afield (including Chirinda and Mt Gorongosa) – are found in the Bvumba area (Table 3). Two of them – Aloe cameronii var. bondana and Aloe inyangensis var. kimberleyana − are varieties of more widespread species and thus of lesser taxonomic significance; both have recently been assessed as Least Concern using IUCN criteria (Timberlake, in press). Of the remaining four taxa, *Barleria fissimuroides* is restricted to just the Bvumba area and a farm just over the border in Mozambique north of Mutare, but three are more widespread, being found from Serra Choa in Mozambique or over the border in Nyanga south to the Himalaya/Tsetserra area or, in the case of *Anthospermum zimbabwense*, to Mt Pene in Chimanimani District. Only one of the near-endemic taxa is threatened, *B. fissimuroides* (Endangered, Darbyshire et al. 2019a). Also of note is the orchid *Angraecum stella-africae*, believed to be extinct in Zimbabwe (Mapaura and Timberlake 2002), but a small colony has been found on the Bvumba in recent years (Wursten 2007). This species has also been found in Malawi (Viphya, Mt Mulanje) and northern South Africa (Wolkberg Mountains) but is nowhere common.

It is surprising that the Bvumba massif has so few endemic or near-endemic taxa, especially when compared to the Chimanimani Mountains (71 endemic taxa, Wursten et al. 2017) or the Nyanga area (19 endemic taxa, revised from Clark et al. 2017). This is obviously not due to under-collection, but possibly the result of the relatively small size of the Bvumba and the preponderance of forest vegetation compared to montane grassland and scrub habitats. Most Nyanga and Chimanimani endemics, for example, are found in montane grassland or montane scrub (Clark et al. 2017; Wursten et al. 2017), habitats poorly represented in the Bvumba and which are also substantially disturbed there.

**Table 3.** Taxa of restricted distribution found in the Bvumba area.

Family/species	Distribution	IUCN Red List assessment
** Asphodelaceae **		
Aloe cameronii *Hemsl.* var. bondana *Reynolds*	Troutbeck, Juliasdale, Bvumba	LC
Aloe inyangensis *Christian* var. kimberleyana *S.Carter*	Nyanga NP, Juliasdale, Stapleford, Bvumba	LC
** Orchidaceae **		
Aeranthes africana *J.L.Stewart*	Bvumba [endemic]	CR D
** Acanthaceae **		
Barleria fissimuroides *I.Darbysh.*	Bvumba, Quinta da Frontiera	EN B2ab
** Gesneriaceae **		
Streptocarpus umtaliensis *B.L.Burtt*	Serra Choa, Nyanga, Stapleford, Bvumba, Tsetserra	LC
** Loranthaceae **		
Englerina oedostemon *(Danser) Polhill & Wiens*	Serra Choa, Nyanga, Stapleford, Mutare, Bvumba, Tsetserra	–
** Rubiaceae **		
Anthospermum zimbabwense *Puff*	Nyanga, Stapleford, Bvumba, Himalaya, Mt Pene	NT B1ab+2ab

### Introduced and invasive species

There are 123 introduced species present in the Bvumba, a result of the diverse human activities there. Although most of these non-native species are benign, a handful are causing major problems. In contrast to other parts of the Manica Highlands, the Bvumba has a high proportion of introduced species that are garden escapes while the rest of the Manica Highlands is affected more by those from commercial forestry.

Invasive woody invasive species on the Bvumba are typically those also found in other parts of the Manica Highlands, and include the Australian *Acacia mearnsii* (wattle) and *A. melanoxylon* (Australian Blackwood) and *Pinus patula*. Australian Blackwood is a particular problem, taking over tracts of open grassland and areas previously under *Protea* cultivation (S. Childes, pers. obs.). Other woody but non-commercial species that have naturalised and have invasive potential (based on evidence elsewhere in the region) are Bauhinia variegata var. variegata, *Cinnamomum camphora*, *C. verum*, *Homalanthus populifolius*, *Jacaranda mimosifolia*, *Psidium cattleianum*, *P. guajava*, *Sambucus canadensis* and *Syzygium jambos*. Classic invasive shrubs are *Lantana camara* and *Solanum mauritianum*, for which there are no easy management solutions, while *Cestrum aurantiacum* is clearly a local problem. A more recent challenge is *Vernonanthura polyanthes* – locally called ‘Beebush’ – which has become rampant since it spread into the Bvumba and Chimanimani areas after its introduction from Brazil to Mozambique (Timberlake et al. 2016b). It spreads rapidly into disturbed and burnt areas that were under wattle, blackwood or eucalyptus (S. Childes. pers. obs.). Clark et al. (2019) postulate that Cyclone Idai (March 2019) might have encouraged the spread of this wind-dispersed species even further afield, although it had earlier also been encountered on the Ribáuè mountains in northern Mozambique (I. Darbyshire, pers. comm. 2017). Indigenous montane forests are being invaded by the garden escapees *Tradescantia fluminensis* and *T. zebrina*, while perhaps the worst forest invader is *Hedychium gardnerianum* – a species that can transform the forest understory and has adverse effects on ground-foraging birds such as Orange Thrush (*Geokichla gurneyi*), Buff-spotted Flufftail (*Sarothrura elegans*) and Cinnamon Dove (*Columba larvata*) that need an open understory with leaf litter (S. Childes, pers. obs.).

### Limitations and future work

Despite the detailed collections, this list is a compilation. As a result, some taxa may have been accidentally omitted whereas others that are found only below 1200 m elevation may have inadvertently been included. In particular, it should be recognised that there are virtually no records from Serra Vumba on the Mozambique side, an area that needs a more detailed collection. Similarly, the drier western slopes of the Bvumba in Zimbabwe have also been undercollected (J. Burrows, pers. comm.). However, with these provisos we estimate that the list is over 90% complete, suggesting a total indigenous flora of around 1250 taxa, of which approximately 1100 would be native flowering plants.

The remaining data gaps for the botanical inventory of the Manica Highlands are centred on the areas immediately to the north and south of the Bvumba, namely Penhalonga-Stapleford and Banti-Himalaya-Tsetserra, respectively. However, some recent survey work has been conducted on Tsetserra and a list of the endemic and range-restricted species has been compiled for that area (J. Osborne, pers. comm.). In contrast to Nyanga, Chimanimani and Bvumba, which had comprehensive available data with which to work, both areas require detailed botanical collecting before reliable lists can be compiled.

## Appendix 1

**Table A1.** Vascular plant checklist for the Bvumba area, E Zimbabwe above 1200 m altitude. Nomenclature follows the Flora of Zimbabwe website (Hyde et al. 2020) with some minor changes. * – introduced species; E – endemic species; NE – near-endemic; FoZ # – Flora of Zimbabwe website record number; s.r. – sight record.

Name / authority	Voucher
PTERIDOPHYTA	
** Anemiaceae **	
Pteris quadriaurita Retz subsp. catoptera (Kunze) Schelpe	Chase 7485
*Mohria lepigera* (Baker) Baker	Chase 3823
*Mohria nudiuscula* J.P.Roux	Chase 7033
*Mohria vestita* Baker	Ballings & Wursten 875
** Aspleniaceae **	
*Asplenium aethiopicum* (Burm.f.) Bech. agg.	Chase 7044
*Asplenium anisophyllum* Kunze	Chase 3471
*Asplenium blastophorum* Hieron.	Müller 3071
*Asplenium boltonii* Brause & Hieron.	Müller 3051
*Asplenium ceii* Pic.Serm.	Ballings & Wursten 39
*Asplenium dregeanum* Kunze	Chase 3457
*Asplenium erectum* Willd.	Ballings & Wursten 40
*Asplenium flexuosum* Schrad.	Chase 3252
*Asplenium formosum* Willd.	Burrows 2151
*Asplenium friesiorum* C.Chr.	Chase 1018
*Asplenium gemmiferum* Schrad.	Ballings s.r.
*Asplenium hypomelas* Kuhn	Chase 7145
*Asplenium inaequilaterale* Willd.	Chase 3516
*Asplenium linckii* Kuhn	Chase 5989
*Asplenium lividum* Kuhn	Chase 6571
*Asplenium lobatum* Pappe & Raws.	Chase 3535
*Asplenium mannii* Hook.	Chase 3526
*Asplenium monanthes* L.	Chase 4663
*Asplenium preussii* Brause	Burrows 1667
*Asplenium protensum* Schrad.	Chase 3514
*Asplenium pumilum* Sw.	Chase 6990
*Asplenium rutifolium* (P.J.Bergius) Kunze	Chase 3137
*Asplenium sandersonii* Hook.	Chase 3490
*Asplenium simii* A.F.Braithw.& Schelpe	Chase 7253
*Asplenium stuhlmannii* Hieron.	Chase 3495
*Asplenium sulcatum* Lam.	Chase 6558
*Asplenium theciferum* (Kunth) Mett.	Ballings & Wursten 19
** Athyriaceae **	
*Athyrium newtonii* Baker	Ballings & Wursten 860
*Athyrium schimperi* Feé	Chase 4407
*Deparia boryana* (Willd.) M.Kato	Ballings & Wursten 857
*Diplazium nemorale* (Baker) Schelpe	Chase 5701
*Diplazium zanzibaricum* (Baker) C.Chr.	Chase 6092
** Blechnaceae **	
*Blechnum attenuatum* (Sw.) Mett.	Chase 3421
*Blechnum tabulare* (Thunb.) Kuhn	Chase 3356
*Cyathea capensis* (L.f.) Sm.	Eyles 3639
*Cyathea dregei* Kunze	Chase 6047
*Cyathea manniana* Hook.	Chase 6696
*Cyathea thomsonii* Baker	Chase 6219
** Davalliaceae **	
*Davallia chaerophylloides* (Poir.) Steud.	Chase 31810
** Dennstaedtiaceae **	
*Blotiella glabra* (Bory) R.M.Tryon	Chase 6275
*Blotiella natalensis* (Hook.) R.M.Tryon	Chase 3364
*Histiopteris incisa* (Thunb.) J.Sm.	Fisher 1303
*Hypolepis sparsisora* (Schrad.) Kuhn	Chase 4585
Pteridium aquilinum (L.) Kuhn subsp. capense (Thunb.) C.Chr.	Wursten & Ballings 1194
** Didymochlaenaceae **	
*Didymochlaena truncatula* (Sw.) J.Sm.	Fisher 1330
** Dryopteridaceae **	
Arachniodes webbiana (A.Braun) Schelpe subsp. foliosa (C.Chr.) Gibby	Ballings & Wursten s.r.
*Ctenitis cirrhosa* (Schumach.) Ching	Ballings & Wursten s.r.
*Dryopteris athamantica* (Kunze) Kuntze	Chase 1048
*Dryopteris kilemensis* (Kuhn) Kuntze	Chase 2034
*Dryopteris manniana* (Hook.) C.Chr.	Chase 6572
*Dryopteris pentheri* (Krasser) C.Chr.	Ballings & Wursten 24
*Megalastrum lanuginosum* (Kaulf.) Holttum	Ballings & Wursten 877
*Nothoperanema squamiseta* (Hook.) Ching	Burrows 1910
*Polystichum transvaalense* N.C.Anthony	Chase 1104
*Polystichum zambesiacum* Schelpe	Chase 7489
** Gleicheniaceae **	
*Dicranopteris linearis* (Burm.f.) Underw.	Ballings 7
** Hymenophyllaceae **	
*Abrodictyum rigidum* (Sw.) Ebihara & Dubuisson	Burrows 2383
*Crepidomanes inopinatum* (Pic.Serm.) J.P.Roux	Ballings & Wursten 853
*Crepidomanes melanotrichum* (Schltdl.) J.P.Roux	Chase 3302
*Didymoglossum erosum* (Willd.) J.P.Roux	Wild 6443
*Hymenophyllum capense* Schrad.	Burrows 2681
*Hymenophyllum kuhnii* C.Chr.	Ballings & Wursten 1623
*Polyphlebium borbonicum* (Bosch.) Ebihara & Dubuisson	Ballings & Wursten 60
** Lomariopsidaceae **	
*Elaphoglossum acrostichoides* (Hook.& Grev.) Schelpe	Chase 6287
*Elaphoglossum aubertii* (Desv.) T.Moore	Chase 4509
*Elaphoglossum kuhnii* Hieron.	Chase 3413
*Elaphoglossum lancifolium* (Desv.) C.V.Morton	Chase 3308
*Elaphoglossum lastii* (Baker) C.Chr.	Chase 118
*Elaphoglossum macropodium* (Fée) T.Moore	Chase 2194
Elaphoglossum spathulatum (Bory) T.Moore var. spathulatum	Burrows 2376
** Lycopodiaceae **	
*Huperzia dacrydioides* (Baker) Pic.Serm.	Chase 1121
*Huperzia ophioglossoides* (Lam.) Rothm.	Chase 1137
*Huperzia verticillata* (L.f.) Trevis.	Chase 3140
*Lycopodiella cernua* (L.) Pic.Serm.	Ballings & Wursten 20
*Lycopodium clavatum* L.	FoZ #4033
** Lygodiaceae **	
*Lygodium kerstenii* Kuhn	Chase 2024
** Marattiaceae **	
Ptisana fraxinea (Sm.) Murdock var. salicifolia (Schrad.) Murdock	Ballings & Wursten 31
** Nephrolepidaceae **	
*Nephrolepis undulata* (Sw.) J.Sm.	Chase 3307
** Oleandraceae **	
*Oleandra distenta* Kunze	Chase 3389
** Ophioglossaceae **	
*Ophioglossum gomezianum* A.Braun	Burrows & Burrows 5193
Ophioglossum polyphyllum A.Braun var. polyphyllum	Ballings & Wursten s.r.
*Ophioglossum reticulatum* L.	Ballings & Wursten 131
** Osmundaceae **	
*Osmunda regalis* L.	Chase 7139
** Polypodiaceae **	
*Belvisia spicata* (L.f.) Copel.	Burrows 2380
*Lepisorus excavatus* (Willd.) Ching	Ballings & Wursten 10
*Lepisorus schraderi* (Mett.) Ching	Ballings & Wursten 14
*Loxogramme abyssinica* (Baker) M.G.Price	Ballings & Wursten 15
*Microgramma mauritiana* (Willd.) Tardieu	Wild & Chase 78
*Microsorum pappei* (Kuhn) Tardieu	Chase 7113
*Pleopeltis macrocarpa* (Willd.) Kaulf.	Ballings & Wursten 18
Pleopeltis polypodioides (L.) E.G.Andrews & Windham subsp. ecklonii (Kunze) J.P.Roux	Ballings & Wursten 80
*Pyrrosia rhodesiana* (C.Chr.) Schelpe	Chase 1038
Pyrrosia schimperiana (Kuhn) Alston var. schimperiana	Wursten & Ballings 81
** Pteridaceae **	
Actiniopteris dimorpha Pic.Serm. subsp. dimorpha	Chase 7578
*Actiniopteris radiata* (Sw.) Link	Chase 5576
*Adiantum capillus*-veneris L.	Ballings & Wursten 1431
*Adiantum poiretii* Wikstr.	Chase 4630
**Adiantum raddianum* C.Presl	Chase 4688
*Pityrogramma argentea* (Willd.) Domin	Chase 3173
*Pteris catoptera* Kunze	Ballings & Wursten 114
*Pteris cretica* L.	Chase 3525
*Pteris dentata* Forssk.	Fisher 1547
*Pteris friesii* Hieron.	Ballings & Wursten 78
*Pteris vittata* L.	Ballings & Wursten 83
Vittaria guineensis Desv. var. orientalis Hieron.	Chase 6641
*Vittaria isoetifolia* Bory	Fisher 1549
*Vittaria volkensii* Hieron.	Ballings & Wursten 144
** Selaginellaceae **	
*Selaginella dregei* (C.Presl) Hieron.	Chase 1126
*Selaginella kraussiana* (Kunze) A.Braun	Chase 4035
*Selaginella mittenii* Baker	Chase 5622
** Sinopteridaceae **	
*Cheilanthes bergiana* Schltdl.	Chase 4591
*Cheilanthes buchananii* (Baker) Domin	Burrows 2324
Cheilanthes involuta (Sw.) Schelpe & N.C.Anthony var. obscura (N.C.Anthony) N.C.Anthony	Chase 3469
*Cheilanthes leachii* (Schelpe) Schelpe	Ballings & Wursten 67
*Cheilanthes multifida* N.C.Anthony & Schelpe	Chase 3979
*Cheilanthes quadripinnata* (Forssk.) Kuhn	Chase 4010
Cheilanthes viridis (Forssk.) Sw. var. glauca (Sim) Schelpe & N.C.Anthony	Ballings & Wursten 68
Cheilanthes viridis (Forssk.) Sw. var. viridis	Chase 7141a
Pellaea calomelanos (Sw.) Link var. calomelanos	Ballings & Wursten 64
Pellaea calomelanos (Sw.) Link var. swynnertoniana (Sim) Schelpe	Chase 4413
*Pellaea doniana* Hook.	Chase 2028
Pellaea dura (Willd.) Hook. var. dura	Chase 5591
*Pellaea pectiniformis* Baker	Chase 4412
** Tectariaceae **	
*Arthropteris monocarpa* (Cordem.) C.Chr.	Chase 3506
Arthropteris orientalis (J.F.Gmel.) Posth. var. orientalis	Chase 2007
*Tectaria gemmifera* (Feé) Alston	Chase 3519
** Thelypteridaceae **	
Amauropelta bergiana (Schltdl.) Holltum var. bergiana	Ballings & Wursten 29
*Christella dentata* (Forssk.) Brownsey & Jermy	Burrows 1636
*Christella gueinziana* (Mett.) Holttum	Chase 3377
*Cyclosorus interruptus* (Willd.) H.Itô	Ballings & Wursten 82
*Pneumatopteris unita* (Kunze) Holttum	Ballings & Wursten 21
*Pseudocyclosorus pulcher* (Willd.) Holttum	Chase 6698
*Thelypteris confluens* (Thunb.) Morton	Ballings & Wursten 23
GYMNOSPERMAE	
** Pinaceae **	
**Pinus patula* Schltdl.& Cham.	FoZ #3770
** Podocarpaceae **	
*Podocarpus milanjianus* Rendle	Chase 5477
MONOCOTYLEDONS	
** Agavaceae **	
**Furcraea foetida* (L.) Haw.	FoZ #40164
** Amaryllidaceae **	
*Boophone disticha* (L.f.) Herb.	FoZ #353
*Crinum macowanii* Baker	FoZ #356
*Cryptostephanus vansonii* I.Verd.	Bamps 711
*Cyrtanthus galpinii* Baker	FoZ #359
**Nothoscordum borbonicum* Kunth	FoZ #15
*Tulbaghia alliacea* (L.f.) Thunb.	Chase 4187
** Anthericaceae **	
*Chlorophytum andongense* Baker	Chase 7321
*Chlorophytum bowkeri* Baker	FoZ #4051
*Chlorophytum comosum* (Thunb.) Jacq.	Jacobsen 3051
*Chlorophytum gallabatense* Baker	Wild 5570
*Chlorophytum galpinii* (Baker) Kativu	FoZ #32168
*Chlorophytum macrosporum* Baker	Chase 1452
** Araceae **	
Zantedeschia albomaculata (Hook.) Baill. subsp. albomaculata	FoZ #34616
** Arecaceae **	
*Phoenix reclinata* Jacq.	FoZ #15154
** Asparagaceae **	
*Asparagus africanus* Lam.	Ferrar 4084
*Asparagus asparagoides* (L.) Wight	FoZ #2885
Asparagus falcatus L. var. falcatus	FoZ #2698
*Asparagus laricinus* Burch.	FoZ #249
*Asparagus setaceus* (Kunth) Jessop	Chase 1623
*Asparagus virgatus* Baker	Müller 3374
** Asphodelaceae **	
*Aloe arborescens* Mill.	FoZ #6111
Aloe cameronii Hemsl. var. bondana Reynolds **NE**	Christian 450
Aloe excelsa A.Berger var. excelsa	FoZ #3451
Aloe inyangensis Christian var. kimberleyana S.Carter **NE**	Plowes 2021
*Aloe pretoriensis* Pole-Evans	Chase 5603
*Aloe rhodesiana* Rendle	FoZ #3817
*Aloe swynnertonii* Rendle	FoZ #3458
*Bulbine latifolia* (L.f.) Roem.& Schult.f.	FoZ #6231
*Kniphofia linearifolia* Baker	FoZ #633
** Behniaceae **	
*Behnia reticulata* (Thunb.) Didr.	Grosvenor 267
** Colchicaceae **	
*Androcymbium striatum* A.Rich.	FoZ #529
*Gloriosa superba* L.	FoZ #1046
** Commelinaceae **	
*Aneilema aequinoctiale* (P.Beauv.) Loudon	FoZ #37419
*Aneilema welwitschii* C.B.Clarke	Bamps 684
*Commelina africana* L.	FoZ #33055
*Commelina welwitschii* C.B.Clarke	FoZ #33108
Cyanotis speciosa (L.f.) Hassk. subsp. speciosa	Chase 609
**Gibasis pellucida* (M.Martens & Galeotti) Hunt	FoZ #18940
*Murdannia simplex* (Vahl) Brenan	Hopkins 7097
**Tradescantia fluminensis* Vell.	FoZ #36272
**Tradescantia zebrina* Bosse	FoZ #40886
** Cyperaceae **	
*Bulbostylis burchellii* (Ficalho & Hiern) C.B.Clarke	Jacobsen 3126
*Bulbostylis hispidula* (Vahl) R.W.Haines	Browning 566
*Bulbostylis schoenoides* (Kunth) C.B.Clarke	FoZ #14528
Carex spicato-paniculata C.B.Clarke	Chase 7425
*Coleochloa setifera* (Ridl.) Gilly	Bamps 606
*Costularia natalensis* C.B.Clarke	Fisher 1643
*Cyperus albostriatus* Schrad.	Chase 8114
*Cyperus amabilis* Vahl	Browning 576
*Cyperus cuspidatus* Kunth	Browning 554
*Cyperus cyperoides* (L.) Kuntze	FoZ #15126
Cyperus denudatus L.f. var. denudatus	Browning 281
*Cyperus distans* L.f.	Jacobsen 3028
*Cyperus hemisphaericus* Boeck.	FoZ #5797
*Cyperus involucratus* Rottb.	Loveridge 1094
*Cyperus pseudoleptocladus* Kük.	Jacobsen 3026
*Cyperus pseudovestitus* (C.B. Clarke) Kük.	Chase 5561
*Cyperus rigidifolius* Steud.	Jacobsen 3023
*Cyperus tenuispica* Steud.	Browning 555
*Cyperus zambesiensis* C.B.Clarke	Müller 3487
Fuirena stricta Steud. var. stricta	Browning 574
*Kyllinga crassipes* Boeck.	Jacobsen 3024
*Kyllinga odorata* Vahl	Jacobsen 3025
*Kyllinga sp*.cf. erecta Schumach.& Thonn.	Hyde s.r.
*Kyllinga squamulata* Vahl	FoZ #6523
*Lipocarpha nana* (A.Rich.) Cherm.	Browning 550
*Mariscus albopilosus* C.B.Clarke	Jacobsen 3047
*Mariscus hemisphaericus* (Boeck.) C.B.Clarke	Jacobsen 3027
*Pycreus pelophilus* (Ridl.) C.B.Clarke	Browning 556
** Dioscoreaceae **	
*Dioscorea dumetorum* (Kunth) Pax	FoZ #19137
*Dioscorea schimperiana* Kunth	Müller 3376
*Dioscorea sylvatica* Eckl.	FoZ #19141
** Dracaenaceae **	
*Dracaena fragrans* (L.) Ker-Gawl.	Chase 1440
*Dracaena mannii* Baker	Chase 875
*Dracaena steudneri* Engl.	Müller plot 76
** Eriocaulaceae **	
*Eriocaulon inyangense* Arw.	Wild 5540
** Eriospermaceae **	
Eriospermum mackenii (Hook.f.) Baker subsp. mackenii	Ferrar s.n.
** Hyacinthaceae **	
*Albuca kirkii* (Baker) Brenan	Ferrar 4040
*Bowiea volubilis* Hook.f.	Chase 362
*Drimia elata* Jacq.	FoZ #7512
Eucomis autumnalis (Mill.) Chitt. subsp. autumnalis (=*E. zambesiaca* Baker)	Plowes 2240
*Ledebouria* unidentified sp.no.1.	FoZ #41510
*Litanthus pusillus* Harv. (=*Drimia uniflora* J.C.Manning & Goldblatt)	FoZ #69141
Stellarioides tenuifolia (F.Delaroche) Speta subsp. tenuifolia (=*Ornithogalum tenuifolium* F.Delaroche)	FoZ #863
** Hydrocharitaceae **	
*Lagarosiphon major* (Ridl.) Moss	Whellan 1561
** Hypoxidaceae **	
*Hypoxis galpinii* Baker	FoZ #15385
*Hypoxis nyasica* Baker	FoZ #53770
*Hypoxis rigidula* Baker	Zimudzi 57
** Iridaceae **	
*Anomatheca grandiflora* Baker (=*Freesia grandiflora* (Baker) Klatt)	FoZ #5749
*Aristea abyssinica* Pax	FoZ #791
*Aristea angolensis* Baker	Garley 119
*Aristea ecklonii* Baker	Chase 63
*Aristea woodii* N.E.Br.	Wild 2845
Crocosmia aurea (Hook.) Planch. subsp. aurea	Chase 5488
*Crocosmia paniculata* (Klatt) Goldblatt	Chase 6315
*Dierama formosum* Hilliard	Chase 6023
*Dietes iridioides* (L.) Klatt	Biegel 2422
*Gladiolus crassifolius* Baker	Plowes 2020
Gladiolus dalenii Van Geel subsp. dalenii	FoZ #796
*Gladiolus flavoviridis* Goldblatt	FoZ #35667
*Hesperantha petitiana* (A.Rich.) Baker	Chase 6036
*Moraea spathulata* (L.f.) Klatt	FoZ #2397
** Juncaceae **	
*Juncus oxycarpus* Kunth	FoZ #40749
** Liliaceae **	
**Lilium formosanum* (Baker) Wallace	FoZ #674
** Musaceae **	
*Ensete ventricosum* (Welw.) Cheesman	FoZ #1060
** Orchidaceae **	
*Aerangis kotschyana* (Rchb.f.) Schltr.	Chase 7083
*Aerangis mystacidii* (Rchb.f.) Schltr.	Chase 5574
*Aeranthes africana* J.L.Stewart **E**	Ball 1283
*Angraecopsis amaniensis* Summerh.	FoZ #1000
*Angraecopsis parviflora* (Thouars) Schltr.	FoZ #34512
*Angraecum chamaeanthus* Schltr.	FoZ #991
*Angraecum conchiferum* Lindl.	Chase 44
*Angraecum minus* Summerh.	Chase 5997
*Angraecum sacciferum* Lindl.	Ferrar 3979
Angraecum stella-africae P.J.Cribb	FoZ #995
Bolusiella iridifolia (Rolfe) Schltr. subsp. picea P.J.Cribb	Wild 2803
*Bonatea steudneri* (Rchb.f.) T.Durand & Schinz	Ball 443
*Brachycorythis lastii* Rolfe	FoZ #4871
Brachycorythis ovata Lindl. subsp. welwitschii (Rchb.f.) Summerh.	Chase 4213
Brachycorythis pleistophylla Rchb.f. subsp. pleistophylla	Chase 5548
*Brownleea maculata* P.J.Cribb	Chase 217
*Brownleea parviflora* Lindl.	Chase 6013
*Bulbophyllum ballii* P.J.Cribb	Chase 6170
*Bulbophyllum elliotii* Rolfe	Wild 2808
Bulbophyllum fuscum Lindl. var. melinostachyum (Schltr.) J.J.Verm.	Wild 2813
*Bulbophyllum josephii* (Kuntze) Summerh.	Wild 2804
*Bulbophyllum longiflorum* Thouars	Ball 1378
*Bulbophyllum maximum* (Lindl.) Rchb.f.	Chase 992
Bulbophyllum sandersonii (Hook.f.) Rchb.f. subsp. sandersonii	Wild 5544
*Bulbophyllum scaberulum* (Rolfe) Bolus	FoZ #1017
Bulbophyllum unifoliatum De Wild. var. infracarinatum (G.Will.) J.J.Verm.	FoZ #1019
*Calanthe sylvatica* (Thouars) Lindl.	Chase 7333
Cynorkis anacamptoides Kraenzl. var. anacamptoides	FoZ #34629
*Cynorkis debilis* (Hook.f.) Summerh. (=*C. hanningtonii* Rolfe)	Chase 5541
*Cynorkis kassneriana* Kraenzl.	Wild 2798
*Cynorkis kirkii* Rolfe	FoZ #99847
Cyrtorchis arcuata (Lindl.) Schltr. subsp. arcuata	Ball 1423
Cyrtorchis praetermissa Summerh. subsp. praetermissa	FoZ #33087
*Cyrtorchis ringens* (Rchb.f.) Summerh.	FoZ #2478
*Diaphananthe fragrantissima* (Rchb.f.) Schltr.	FoZ #21698
*Diaphananthe rutila* (Rchb.f.) Summerh.	Wild 6446
*Diaphananthe stolzii* Schltr.	Ball 1303
*Diaphananthe subsimplex* Summerh.	Ball 1406
*Diaphananthe xanthopollinia* (Rchb.f.) Summerh.	Ball 1236
Disa aconitoides Sond. subsp. concinna (N.E.Br.) H.P.Linder	Chase 6268
Disa fragrans Schltr. subsp. fragrans	Chase 4065
*Disperis anthoceros* Rchb.f.	Wild 2816
*Disperis dicerochila* Summerh.	FoZ #2374
*Disperis lindleyana* Rchb.f.	Chase 7030
*Disperis virginalis* Schltr.	Wild 2810
*Eulophia callichroma* Rchb.f.	Symoens et al. 669
*Eulophia cucullata* (Sw.) Steud.	FoZ #4873
*Eulophia eylesii* Summerh.	Chase 5562
*Eulophia fridericii* (Rchb.f.) A.V.Hall	Ball 440
*Eulophia gonychila* Schltr.	Chase 5572
Eulophia hians Spreng. var. hians	FoZ #21961
*Eulophia horsfallii* (Bateman) Summerh.	Ball 5256
*Eulophia norlindhii* Summerh.	Wild s.n.
*Eulophia nyasae* Rendle	Ball 717
*Eulophia parviflora* (Lindl.) A.V.Hall	Chase 3075
*Eulophia petersii* (Rchb.f.) Rchb.f.	Chase 51
*Eulophia rolfeana* Kraenzl. (=*E. williamsonii* P.J.Cribb)	Beasley 121
*Eulophia speciosa* (Lindl.) Bolus	Chase 4180
*Eulophia streptopetala* Lindl.	FoZ #3623
*Eulophia tenella* Rchb.f.	Wild 6746
*Eulophia venulosa* Rchb.f.	Ball 586
*Habenaria amoena* Summerh.	Ballings s.r.
*Habenaria armatissima* Rchb.f.	Ball 442
*Habenaria cornuta* Lindl.	Ball 522
*Habenaria galpinii* Bolus	Ball 528
*Habenaria macrostele* Summerh.	Chase 6050
*Habenaria malacophylla* Rchb.f.	Chase 4403
Habenaria nyikana Rchb.f. subsp. nyikana	FoZ #7002
Habenaria praestans Rendle var. praestans	Chase 206
*Habenaria rautaneniana* Kraenzl.	Chase 6348
*Habenaria silvatica* Schltr.	Chase 6334
*Habenaria subaequalis* Summerh.	Wild 2806
*Jumellea walleri* (Rolfe) la Croix	Wild 3223
*Liparis bowkeri* Harv.	Grosvenor 784
*Liparis mulindana* Schltr.	Ball 1267
*Liparis nervosa* (Thunb.) Lindl.	FoZ #1411
*Malaxis weberbaueriana* (Kraenzl.) Summerh. (=*M. stolzii* (Schltr.) Summerh.)	Chase 6363
*Microcoelia exilis* Lindl.	Ferrar s.n.
*Microcoelia globulosa* (Ridl.) L.Jonss.	FoZ #5781
*Microcoelia stolzii* (Schltr.) Summerh.	Ball 1392
*Mystacidium tanganyikense* Summerh. (incl. *M. pusillum* sensu Wild 6442)	FoZ #2455
*Nervilia ballii* G.Will.	Ball 585
*Nervilia crociformis* (Zoll.& Moritzi) Seidenf.	Ball 1407
Nervilia kotschyi (Rchb.f.) Schltr. var. purpurata (Rchb.f.& Sond.) Börge Pett.	Ball 511
*Nervilia pectinata* P.J.Cribb	Ball 410
*Nervilia shirensis* (Rolfe) Schltr.	Chase 6235
*Orthochilus eustachyus* (Rchb.f.) Bytebier	FoZ #2854
*Orthochilus mechowii* Rchb.f.	FoZ #102482
*Orthochilus milnei* (Rchb.f.) Bytebier (=*Eulophia milnei* Rchb.f.)	Jacobsen 3030
*Orthochilus odontoglossus* (Rchb.f.) Bytebier	FoZ #37451
*Platycoryne pervillei* Rchb.f.	FoZ #4075
*Polystachya adansoniae* Rchb.f.	Chase 5609
Polystachya albescens Ridl. subsp. imbricata (Rolfe) Summerh.	Drummond 5097
Polystachya caespitifica Engl. subsp. hollandii (Bolus) P.J.Cribb & Podz.	FoZ #36207
*Polystachya campyloglossa* Rolfe [incl. *P. ottoniana* sensu Ball 1282, Chase 86]	Ball 1282
*Polystachya concreta* (Jacq.) Garay & H.R.Sweet (=*P. tessellata* Lindl.)	Chase 4400
*Polystachya cultriformis* (Thouars) Spreng.	Grosvenor 659
*Polystachya fusiformis* (Thouars) Lindl.	FoZ #1659
*Polystachya golungensis* Rchb.f.	Ball 1329
*Polystachya modesta* Rchb.f.	FoZ #15642
*Polystachya simplex* Rendle	Ball 1377
*Polystachya stuhlmannii* Kraenzl.	Philcox et al. 8959
*Polystachya subumbellata* P.J.Cribb & Podz.	Wild 6445
*Polystachya transvaalensis* Schltr.	FoZ #1667
*Polystachya vaginata* Summerh.	Wild 2977
*Polystachya zambesiaca* Rolfe	Ball 1364
*Rangaeris muscicola* (Rchb.f.) Summerh.	Plowes 2893
*Satyrium anomalum* Schltr.	FoZ #1415
*Satyrium chlorocorys* Rolfe	Chase 6014
*Satyrium longicauda* Lindl.	Hopkins 7077
*Satyrium trinerve* Lindl.	FoZ #34622
*Solenangis conica* (Schltr.) L.Jonss.	FoZ #3052
*Stenoglottis woodii* Schltr.	Woodburn 30b
*Stenoglottis zambesiaca* Rolfe (incl. *S. fimbriata* sensu Summerh.)	Chase 6018
Stolzia repens (Rolfe) Summerh. var. obtusa G.Will.	Ball 1398
Stolzia repens (Rolfe) Summerh. var. repens	Wild 2811
*Taeniophyllum coxii* (Summerh.) Summerh.	FoZ #64342
*Tridactyle anthomaniaca* (Rchb.f.) Summerh.	Grosvenor 862
*Tridactyle bicaudata* (Lindl.) Schltr.	Plowes 2892
*Tridactyle inaequilonga* (De Wild.) Schltr.	Obermeyer 2087
*Tridactyle tricuspis* (Bolus) Schltr.	Chase 6101
*Tridactyle tridactylites* (Rolfe) Schltr.	Ball 160
*Tridactyle tridentata* (Harv.) Schltr.	FoZ #15348
*Vanilla polylepis* Summerh.	Plowes 2667
*Ypsilopus erectus* (P.J.Cribb) P.J.Cribb & J.L.Stewart	Ball 1315
** Poaceae **	
*Agrostis lachnantha* Nees	Corby 244
Andropogon eucomus Nees subsp. eucomus	FoZ #15338
Andropogon eucomus Nees subsp. huillensis (Rendle) Sales	Hyde s.r.
*Andropogon schirensis* A.Rich.	Pope et al. 6608
*Arthraxon lancifolius* (Trin.) Hochst.	Chase 7096
*Bewsia biflora* (Hack.) Gooss.	Pope et al 6606
*Brachypodium flexum* Nees	Hyde 97
**Briza maxima* L.	FoZ #93
**Briza minor* L.	FoZ #95
**Bromus catharticus* Vahl	Hyde 297
**Cenchrus clandestinus* (Chiov.) Morrone (=*Pennisteum clandestinum* Chiov.)	FoZ #4006
*Coelachne africana* Pilg.	Biegel 2427
*Craspedorhachis africana* Benth.	Runhaar 844
*Cymbopogon nardus* (L.) Rendle	Runhaar 77
*Cynodon dactylon* (L.) Pers.	Hyde s.r.
*Digitaria diagonalis* (Nees) Stapf	Rattray 1460
*Digitaria eriantha* Steud.	Cleghorn 1360
*Digitaria gazensis* Rendle	Hyde s.r.
*Digitaria maitlandii* Stapf & C.E.Hubb.	Fisher 1127
*Digitaria scalarum* (Schweinf.) Chiov.	Allison s.n.
Diheteropogon amplectens (Nees) Clayton var. catangensis (Chiov.) Clayton	FoZ #15215
*Ehrharta erecta* Lam.	Corby 247
*Eleusine africana* Kenn.-O’Byrne	FoZ #16428
*Elionurus muticus* (Spreng.) Kuntze	Corby 249
*Eragrostis acraea* De Winter	Crook P70
*Eragrostis capensis* (Thunb.) Trin.	Runhaar 840
*Eragrostis chapelieri* (Kunth) Nees	Fisher 220
*Eragrostis cilianensis* (All.) Janch.	Crook 1050
*Eragrostis congesta* Oliv.	Corby 262
*Eragrostis hispida* K.Schum.	FoZ #15208
*Eragrostis patens* Oliv.	Runhaar 761
*Eragrostis plana* Nees	Wild 2
*Eragrostis racemosa* (Thunb.) Steud.	Corby 261
Eragrostis sclerantha Nees subsp. villosipes (Jedwabn.) Launert	Runhaar 757
*Eragrostis tenuifolia* (A.Rich.) Steud.	FoZ #15225
*Heteropogon contortus* (L.) Roem.& Schult.	Hyde s.r.
*Hyparrhenia anamesa* Clayton	Hyde 345
*Hyparrhenia cymbaria* (L.) Stapf	Hyde 350
*Hyparrhenia filipendula* (Hochst.) Stapf	Hyde s.r.
Hyparrhenia newtonii (Hack.) Stapf var. macra Stapf	Corby 246
Hyparrhenia newtonii (Hack.) Stapf var. newtonii	Runhaar 762
*Hyparrhenia nyassae* (Rendle) Stapf	Runhaar 764
*Imperata cylindrica* (L.) Raeusch.	FoZ #37404
*Isachne mauritiana* Kunth	Crook 2016
*Ischaemum fasciculatum* Brongn.	Runhaar 767
*Koeleria capensis* (Steud.) Nees	Corby 268
**Lolium multiflorum* Lam.	Corby 269
*Loudetia flavida* (Stapf) C.E.Hubb.	Rattray 1457
*Loudetia simplex* (Nees) C.E.Hubb.	FoZ #16425
Melinis ambigua Hack. subsp. ambigua	Runhaar 838
*Melinis minutiflora* P.Beauv.	Fisher 1596
*Melinis nerviglumis* (Franch.) Zizka	Hyde 295
Melinis repens (Willd.) Zizka subsp. grandiflora (Hochst.) Zizka	Schweickert 227
*Microchloa caffra* Nees	Corby 264
*Monocymbium ceresiiforme* (Nees) Stapf	Hyde 72
*Olyra latifolia* L.	Müller 3381
*Oplismenus burmannii* (Retz.) P.Beauv.	Pope 3728
*Oplismenus compositus* (L.) P.Beauv.	Müller 3047
*Oplismenus hirtellus* (L.) P.Beauv.	Rattray 1449
*Oplismenus undulatifolius* (Ard.) Roem.& Schult.	Runhaar 752a
*Oxytenanthera abyssinica* (A.Rich.) Munro	Chase 2172
*Panicum inaequilatum* Stapf & C.E.Hubb.	Crook 2020
*Panicum laticomum* Nees	Müller s.n.
*Panicum maximum* Jacq.	Rattray 1458
*Panicum monticola* Hook.f.	Runhaar 755
*Panicum wiehei* Renvoize	Drummond 5094
**Paspalum dilatatum* Poir.	Hyde s.r.
*Paspalum scrobiculatum* L.	FoZ #15125
**Paspalum urvillei* Steud.	FoZ #2054
*Perotis patens* Gand.	Wild 5
**Poa annua* L.	Hyde 103
*Poecilostachys oplismenoides* (Hack.) Clayton (=*Chloachne oplismenoides* (Hack.) Robyns)	Chase 1144
*Pogonarthria squarrosa* (Roem.& Schult.) Pilg.	Hyde s.r.
*Pseudechinolaena polystachya* (Kunth) Stapf	Runhaar 842
*Rhytachne rottboellioides* Desv.	Hyde s.r.
*Sacciolepis typhura* (Stapf) Stapf	Chase 7836
*Schizachyrium sanguineum* (Retz.) Alston	Pope 6607
*Setaria homonyma* (Steud.) Chiov.	FoZ #33050
*Setaria megaphylla* (Steud.) T.Durand & Schinz	Müller 3078
*Setaria sphacelata* (Schumach.) Moss	Wild 1
*Setaria verticillata* (L.) P.Beauv.	FoZ #2086
*Sporobolus acinifolius* Stapf	Hyde 94
*Sporobolus molleri* Hack.	Hyde s.r.
*Sporobolus piliferus* (Trin.) Kunth	Hyde s.r.
*Sporobolus pyramidalis* P.Beauv.	FoZ #15122
*Sporobolus sanguineus* Rendle	Hyde 135
*Stereochlaena cameronii* (Stapf) Pilg.	Hyde s.r.
*Streblochaete longiarista* (A.Rich.) Pilg.	Müller 3377
*Themeda triandra* Forssk.	FoZ #15216
*Tragus berteronianus* Schult.	Crook 1052
*Tricholaena monachne* (Trin.) Stapf & C.E.Hubb.	FoZ #15211
*Tristachya nodiglumis* K.Schum.	Chase 7835
*Urochloa oligotricha* (Fig.& De Not.) Henrard	Sheppard 43
** Potamogetonaceae **	
*Potamogeton nodosus* Poir.	FoZ #7454
*Potamogeton octandrus* Poir.	Chase 5981
*Potamogeton pusillus* L.	Denny 1315
** Smilaceae **	
*Smilax anceps* Willd.	Wild 496
** Strelitziaceae **	
*Strelitzia caudata* R.A.Dyer	FoZ #1208
** Typhaceae **	
*Typha capensis* (Rohrb.) N.E.Br.	FoZ #99860
** Velloziaceae **	
*Xerophyta* sp.	Hyde s.r.
** Xyridaceae **	
*Xyris obscura* N.E.Br.	Chase 6027
** Zingiberaceae **	
*Aframomum albiflorum* Lock	FoZ #39633
*Aframomum angustifolium* (Sonn.) K.Schum.	FoZ #34511
**Hedychium gardnerianum* Ker Gawl.	FoZ #294
*Siphonochilus aethiopicus* (Schweinf.) B.L.Burtt	Eyles 6962
DICOTYLEDONS	
** Acanthaceae **	
*Anisotes pubinervis* (T.Anderson) Heine (=*Metarungia pubinervia* (T.Anderson) C.B.Clarke)	Chase 8161
Asystasia gangetica (L.) T.Anderson subsp. micrantha (Nees) Ensermu	Chase 2154
*Barleria aromatica* Oberm.	Hopkins 8049
*Barleria fissimuroides* I.Darbysh. **NE**	Chase s.n.
Barleria spinulosa Klotzsch subsp. kirkii (T.Anderson) I.Darbysh.	Chase 6495
*Barleria ventricosa* Nees	FoZ #24678
*Brillantaisia cicatricosa* Lindau (=*B. ulugurica* Lindau)	Chase 854
*Dicliptera clinopodia* Nees	FoZ #1238
*Dicliptera extenta* S.Moore	Müller 3042
*Dyschoriste nagchana* (Nees) Bennet	FoZ #33058
Dyschoriste trichocalyx (Oliv.) Lindau subsp. verticillaris (C.B.Clarke) Vollesen (=*D. verticillaris* C.B.Clarke)	Carter 2123
*Hypoestes aristata* (Vahl) Roem.& Schult.	Carter 2119
Hypoestes forskaolii (Vahl) Roem.& Schult. subsp. forskaolii	Chase 6493
**Hypoestes phyllostachya* Baker	FoZ #36366
*Isoglossa gregorii* (S.Moore) Lindau	Müller 3404
*Isoglossa milanjiensis* S.Moore (=*I. mossambicensis* Lindau)	Chase 7104
*Justicia betonica* L.	Chase 2159
*Justicia bracteata* (Hochst.) Zarb (=*Monochema debile* (Forssk.) Nees)	Chase 4557
*Justicia matammensis* (Schweinf.) Oliv.	Wild 2838
*Justicia nyassana* Lindau	Chase 5578
*Justicia phyllostachys* C.B.Clarke	FoZ #15150
*Justicia striata* (Klotzsch) Bullock	FoZ #28977
*Mellera lobulata* S.Moore	Chase 5721
*Mimulopsis solmsii* Schweinf.	FoZ #1616
Phaulopsis imbricata (Forssk.) Sweet subsp. imbricata	Chase 786
*Pseuderanthemum subviscosum* (C.B.Clarke) Stapf	Chase 1785
*Ruellia cordata* Thunb.	FoZ #33086
*Sclerochiton harveyanus* Nees	Hopkins 7093
*Thunbergia alata* Sims	Obermeyer 2141
*Thunbergia natalensis* Hook.	Biegel 2421
*Thunbergia oblongifolia* Oliv. (incl. *T. lancifolia* sensu Mapaura & Timberlake)	FoZ #3602, Chase 4689
*Thunbergia petersiana* Lindau	Grosvenor 783
*Thunbergia usambarica* Lindau	FoZ #228
** Achariaceae **	
*Kiggelaria africana* L.	Chase 7797
*Rawsonia lucida* Harv.& Sond.	Fisher 1513
** Amaranthaceae **	
*Achyranthes aspera L. var. pubescens (Moq.) C.C.Towns.	Müller 3090
*Achyranthes aspera L. var. sicula L.	Chase 2151
**Alternanthera caracasana* Kunth	FoZ #15032
**Amaranthus hybridus* L.	FoZ #37392
Amaranthus lividus L. subsp. polygonoides (Moq.) Probst	FoZ #15033
*Centemopsis gracilenta* (Hiern) Schinz	FoZ #4876
**Chenopodium ambrosioides* L.	FoZ #41453
**Chenopodium album* L.	FoZ #25901
*Cyathula cylindrica* Moq.	Schelpe 363
*Cyathula uncinulata* (Schrad.) Schinz	FoZ #301
**Gomphrena celosioides* Mart.	FoZ #33043
** Anacardiaceae **	
Lannea edulis (Sond.) Engl. var. edulis	FoZ #15148
**Mangifera indica* L.	FoZ #3612
*Searsia chirindensis* (Baker f.) Moffett (=*Rhus chirindensis* Baker f.)	Müller 3596
*Searsia dentata* (Thunb.) F.A.Barkley (=*Rhus dentata* Thunb.)	Müller 806
*Searsia longipes* (Engl.) Moffett var. longipes (=Rhus longipes Engl. var. longipes)	Loveridge 1090
*Searsia lucida* (L.) F.A.Barkley (=*Rhus lucida* L.)	Chase 7358
*Searsia natalensis* (C.Krauss) F.A.Barkley (=*Rhus natalensis* C.Krauss)	Müller 3619
*Searsia tomentosa* (L.) F.A.Barkley (=*Rhus tomentosa* L.)	FoZ #26271
** Annonaceae **	
Annona senegalensis Pers. subsp. senegalensis	FoZ #15212
*Artabotrys monteiroae* Oliv.	Chase 5822
*Monanthotaxis chasei* (N.Robson) Verdc.	Müller 3075
Uvaria lucida Benth. subsp. virens (N.E.Br.) Verdc.	Müller 3094
*Xylopia parviflora* (A.Rich.) Benth.	Müller 3618
** Aphloiaceae **	
*Aphloia theiformis* (Vahl) Benn.	Chase 517
** Apiaceae **	
*Alepidea peduncularis* A.Rich.	FoZ #7647
**Apium leptophyllum* (Pers.) Benth.	FoZ #98903
*Centella asiatica* (L.) Urb.	FoZ #24588
Centella virgata (L.f.) Drude var. gracilescens Domin	Chase 6116
Diplolophium buchananii (Oliv.) C.Norman subsp. swynnertonii (Baker f.) Cannon	Symoens et al. 667
Heteromorpha arborescens (Spreng.) Cham.& Schltdl. var. abyssinica (A.Rich.) H.Wolff	Chase 4417
Heteromorpha arborescens (Spreng.) Cham.& Schltdl. var. montana P.J.D.Winter	FoZ #35269
*Hydrocotyle mannii* Hook.f.	Wild 2840
*Pimpinella caffra* (Eckl.& Zeyh.) D.Dietr.	Chase 6032
*Sanicula elata* D.Don	FoZ #23850
Steganotaenia araliacea Hochst. var. araliacea	FoZ #33124
** Apocynaceae **	
Asclepias cucullata (Schltr.) Schltr. subsp. scabrifolia (S.Moore) Goyder	Wursten & Ballings 211
Carissa bispinosa (L.) Brenan subsp. zambesiensis Kupicha	Chase 7018
*Carissa spinarum* L. (=*C. edulis* (Forssk.) Vahl)	FoZ #98852
*Ceropegia abyssinica* Decne.	Chase 5535
*Ceropegia lugardae* N.E.Br.	Chase 1234
*Cryptolepis oblongifolia* Schltr.	FoZ #31948
*Cynanchum umtalense* Liede (=*Cynanchum* sp. no.1)	Chase 6465
*Glossostelma carsonii* (N.E.Br.) Bullock	Chase 66
Huernia hislopii Turrill subsp. hislopii	Wild 2805
*Landolphia buchananii* (Hallier f.) Stapf	Chase 4594
*Landolphia kirkii* Hook.f.	FoZ #33147
Margaretta rosea Oliv. subsp. whytei (K.Schum.) M.Mwanyambo	FoZ #15142
*Mondia whitei* (Hook.f.) Skeels	FoZ #2472
*Pachycarpus chirindensis* (S.Moore) Goyder	Obermeyer 2113
*Rauvolfia caffra* Sond.	Bamps 587
*Secamone alpini* Schult.	Pole Evans 5114
*Sphaerocodon caffrum* (Meisn.) Schltr.	Chase 5563
*Strophanthus speciosus* (Ward & Harv.) Reber	FoZ #2873
*Tabernaemontana stapfiana* Britten	Biegel 2418
*Tabernaemontana ventricosa* A.DC.	Drummond 5082
*Tylophora anomala* N.E.Br. (=*Cynanchum chirindense* S.Moore)	FoZ #28705
Tylophora sp.no. 2 cf. tenuipedunculata (Müller 2841)	Harder 3817
** Aquifoliaceae **	
Ilex mitis (L.) Radlk. var. mitis	Chase 3084
** Araliaceae **	
*Cussonia arborea* A.Rich.	Bamps 634
*Cussonia spicata* Thunb.	FoZ #1163
*Polyscias fulva* (Hiern) Harms	Chase 1971
*Schefflera goetzenii* Harms	Wild 2837
*Schefflera umbellifera* (Sond.) Baill.	Chase 5591
** Aristolochiaceae **	
*Aristolochia albida* Duch.	Pole Evans 4822
** Asteraceae **	
**Acanthospermum australe* (Loefl.) Kuntze	FoZ #433
*Adenostemma mauritianum* DC.	Müller 1950
**Ageratum conyzoides* L.	FoZ #3545
**Ageratum houstonianum* Mill.	FoZ #435
Anisopappus chinensis (L.) Hook.f.& Arn. subsp. chinensis var. dentatus (DC.) S.Ortíz, Paiva & Rodr.Oubiña	Chase 5581
*Anisopappus kirkii* (Oliv.) Brenan	Schelpe 378
*Aspilia mossambicensis* (Oliv.) Wild	FoZ #4882
Aspilia pluriseta Schweinf. subsp. pluriseta	Bamps et al. 640
Athrixia rosmarinifolia (Walp.) Oliv.& Hiern var. rosmarinifolia	Loveridge 1089
*Berkheya setifera* DC.	FoZ #39978
*Berkheya zeyheri* (Sond.& Harv.) Oliv.& Hiern	FoZ #3926
**Bidens biternata* (Lour.) Merr.& Sherff	Chase 2153
**Bidens pilosa* L.	FoZ #443
Bothriocline inyangana N.E.Br. var. inyangana	Hopkins 6935
*Centratherum punctatum Cass. subsp. punctatum	FoZ #6295
Chrysanthemoides monilifera (L.) Norl. subsp. septentrionale Norl.	FoZ #3596
*Cineraria deltoidea* Sond.	Chase 7787
*Cineraria pulchra* Cron	FoZ #21501
**Conyza aegyptiaca* (L.) Aiton	FoZ #37464
**Conyza bonariensis* (L.) Cronquist	FoZ #19057
*Conyza gouanii* (L.) Willd.	FoZ #2258
*Conyza pinnata* (L.f.) Kuntze	FoZ #7645
**Conyza sumatrensis* (Retz.) E.Walker	FoZ #2471
**Coreopsis lanceolata* L.	FoZ #489
**Cotula australis* (Sieb.& Spreng.) Hook.f.	FoZ #933
*Crassocephalum crepidioides* (Benth.) S.Moore	Chase 8303
Crassocephalum rubens (Jacq.) S.Moore var. rubens	FoZ #447
Crassocephalum rubens (Jacq.) S.Moore var. sarcobasis (DC.) C.Jeffrey & Beentje	Chase 4576
Crassocephalum × picridifolium (DC.) S.Moore	Chase 2148
Dichrocephala integrifolia (L.f.) Kuntze subsp. integrifolia	Chase 7391
*Emilia coccinea* (Sims) G.Don	FoZ #4114
*Emilia discifolia* (Oliv.) C.Jeffrey	FoZ #7655
**Erigeron karvinskianus* DC.	FoZ #3551
*Erythrocephalum zambesianum* Oliv.& Hiern	Wild 469
**Euryops chrysanthemoides* (DC.) B.Nord.	FoZ #41177
**Galinsoga parviflora* Cav.	FoZ #7662
**Galinsoga quadriradiata* Ruiz & Pav.	FoZ #7665
*Gerbera piloselloides* (L.) Cass.	Chase 8421
Gerbera viridifolia (DC.) Sch.Bip. subsp. viridifolia	FoZ #651
**Gnaphalium purpureum* L.	FoZ #2248
Gutenbergia cordifolia Oliv. var. marginata (O.Hoffm.) C.Jeffrey	Chase 8291
Helichrysum adenocarpum DC. subsp. adenocarpum	Bacon 6844
*Helichrysum asperum* (Thunb.) Hilliard & B.L.Burtt	FoZ #21420
*Helichrysum buchananii* Engl.	Chase 6114
*Helichrysum candolleanum* H.Buek	Carter et al. 2121
*Helichrysum cephaloideum* DC.	Chase 6026
*Helichrysum goetzeanum* O.Hoffm.	FoZ #41358
*Helichrysum kilimanjari* Oliv.	Symoens et al. 687
*Helichrysum kraussii* Sch.Bip.	Bishop 394
*Helichrysum lepidissimum* S.Moore	Chase 6115
Helichrysum nitens Oliv.& Hiern subsp. nitens	Bacon 6843
Helichrysum nudifolium (L.) Less. var. nudifolium	FoZ #4883
Helichrysum nudifolium (L.) Less. var. oxyphyllum (DC.) Beentje	FoZ #4884
Helichrysum nudifolium (L.) Less. var. pilosellum (L.f.) Beentje	Wild 468
*Helichrysum odoratissimum* (L.) Sweet	FoZ #2344
Helichrysum panduratum O.Hoffm. var. panduratum	Chase 1237
Helichrysum panduratum O.Hoffm. var. transvaalense Moeser	FoZ #7468
*Helichrysum schimperi* (A.Rich.) Moeser	Chase 1237
*Helichrysum umbraculigerum* Less.	Chase 1355
*Hypericophyllum compositarum* Steetz	Chase 7093
**Hypochaeris radicata* L.	FoZ #2048
*Inula glomerata* Oliv.& Hiern	FoZ #1413
*Kleinia chimanimaniensis* Van Jaarsv. (=*K. galpinii* Hook.f.)	Schelpe 377
*Lactuca inermis* Forssk.	FoZ #19054
*Laggera crispata* (Vahl) Hepper & J.R.I.Wood	FoZ #33120
Lipotriche scandens (Schumach.) Orchard subsp. madagascariensis (Baker) D.J.N.Hind (=*Melanthera scandens* (Schumach.& Thonn.) Roberty)	Chase 7111
*Microglossa pyrifolia* (Lam.) Kuntze	FoZ #1621
*Mikania carteri* Baker	FoZ #4007
*Mikania* sp. of FoZ	Müller 3036
*Mikaniopsis cissampelina* (DC.) C.Jeffrey	Fisher 1576
**Montanoa hibiscifolia* Benth.	Wild 5513
**Neojeffreya decurrens* (L.) Cabrera	FoZ #7332
Nidorella auriculata DC. subsp. auriculata	FoZ #15227
Nidorella resedifolia DC. subsp. resedifolia	Chase 761
*Osteospermum monocepahalum* (Oliv.& Hiern) Norl.	FoZ #3919
*Phymaspermum bolusii* (Hutch.) Källersjö	FoZ #15330
**Pseudognaphalium luteo-album* (L.) Hilliard & B.L.Burtt	FoZ #2186
*Pseudognaphalium oligandrum* (DC.) Hilliard & B.L.Burtt	FoZ #2188
*Schistostephium crataegifolium* (DC.) Harv. (=*S. artemisiifolium* Baker)	Bishop 395
*Schistostephium heptalobum* (DC.) Oliv.& Hiern	FoZ #15218
*Schistostephium oxylobum* S.Moore	Chase 8423
*Senecio deltoideus* Less.	FoZ #2219
*Senecio gazensis* S.Moore	FoZ #15411
*Senecio hochstetteri* A.Rich.	Wild 2821
*Senecio inornatus* DC.	Chase 6105
*Senecio latifolius* DC.	Wild 38
**Senecio macroglossus* DC.	Fisher 1545
*Senecio milanjianus* S.Moore	Wild 2830
*Senecio oxyriifolius* DC.	Wild 520
*Senecio purpureus* L.	Chase 7140
*Senecio ruwenzoriensis* S.Moore	Wild 2822
*Senecio syringifolius* O.Hoffm.	Hopkins 8046
*Senecio tamoides* DC.	FoZ #2222
*Senecio triactinus* S.Moore	FoZ #5793
*Sigesbeckia orientalis* L.	FoZ #84888
*Solanecio mannii* (Hook.f.) C.Jeffrey	FoZ #469
Sonchus friesii Boulos var. integer G.V.Pope	FoZ #922
**Sonchus oleraceus* L.	FoZ #920
*Spilanthes mauritiana* (Pers.) DC.	Eyles 7082
*Stomatanthes africanus* (Oliv.& Hiern) R.M.King & H.Rob.	FoZ #15199
**Tagetes minuta* L.	FoZ #35492
*Tolpis capensis* (L.) Sch.Bip.	FoZ #3753
**Tridax procumbens* L.	FoZ #3925
**Vernonanthura polyanthes* (Sprengel) Vega & Dematteis	FoZ #3740
*Vernonia acuminatissima* S.Moore	Hopkins 8053
*Vernonia adoensis* Sch.Bip.	FoZ #25856
Vernonia bainesii Oliv.& Hiern subsp. bainesii	Whellan 747
Vernonia calvoana (Hook.f.) Hook.f. subsp. meridionalis (Wild) C.Jeffrey	FoZ # 475
Vernonia colorata (Willd.) Drake subsp. colorata	FoZ #2322
*Vernonia galpinii* Klatt	Chase 3105
*Vernonia glaberrima* O.Hoffm.	FoZ #4878
*Vernonia holstii* O.Hoffm.	Chase 5595
*Vernonia karaguensis* Oliv.& Hiern	Chase 7138
*Vernonia lundiensis* (Hutch.) Wild & G.V.Pope	Ferrar 4069
Vernonia melleri Oliv.& Hiern var. melleri	Chase 5582
*Vernonia myriantha* Hook.f.	Plowes 2706
*Vernonia natalensis* Walp.	Fisher 212
*Vernonia wollastonii* S.Moore	Müller 3060
*Vernonia petersii* Oliv.	FoZ #43356
** Balsaminaceae **	
Impatiens cecilii N.E.Br. subsp. cecilii	Plowes 2322
*Impatiens sylvicola* Burtt Davy & Greenway	Eyles 5476
** Begoniaceae **	
*Begonia sonderiana* Irmsch.	Chase 995
** Bignoniaceae **	
**Amphilophium crucigerum* (L.) L.G.Lohmann	FoZ #18307
**Dolichandra unguis*-cati (L.) L.G.Lohmann	FoZ #3975
**Jacaranda mimosifolia* D.Don	FoZ #18352
*Podranea brycei* (N.E.Br.) Sprague	FoZ #3576
** Boraginaceae **	
*Cynoglossum lanceolatum* Forssk.	Chase 4390
*Cynoglossum wildii* E.S.Martins	Williams 215
** Brassicaceae **	
*Cardamine africana* L.	FoZ #15459
**Cardamine flexuosa* With.	FoZ #636
**Coronopus didymus* (L.) Sm.	FoZ #19047
**Lepidium bonariense* L.	FoZ #41218
** Cactaceae **	
*Rhipsalis baccifera* (J.Mill.) Stearn	Eyles 6589
** Campanulaceae **	
*Lobelia erinus* L.	Schelpe 366
*Lobelia goetzei* Diels	FoZ #867
*Lobelia stricklandiae* Gilliland	Wild 1592
*Wahlenbergia denticulata* (Burch.) A.DC.	FoZ #34881
*Wahlenbergia madagascariensis* A.DC.	FoZ #5012
Wahlenbergia subaphylla (Baker) Thulin subsp. scoparia (Wild) Thulin	Chase 3104
*Wahlenbergia undulata* (L.f.) A.DC.	Chase 6125
*Wahlenbergia virgata* Engl.	FoZ #3750
** Capparaceae **	
*Cleome monophylla* L.	Hyde s.r.
*Ritchiea albersii* Gilg	Chase 6225
** Caprifoliaceae **	
**Sambucus canadensis* L.	FoZ #37446
** Caryophyllaceae **	
**Cerastium glomeratum* Thuill.	Whellan 1560
Drymaria cordata (L.) Roem.& Schult. var. cordata	Chase 6049
**Sagina apetala* Ard.	FoZ #1808
Silene burchellii DC. var. angustifolia Sond.	Chase 7224
*Stellaria mannii* Hook.f.	Müller plot 68
**Stellaria media* (L.) Vill.	FoZ #79972
** Celastraceae **	
*Allocassine laurifolia* (Harv.) N.Robson	Chase 7199
*Catha edulis* (Vahl) Endl.	Chase 784
*Elaeodendron croceum* (Thunb.) DC.	Müller plot 150
*Gymnosporia buxifolia* (L.) Szyszyl.	Chase 5571
Gymnosporia harveyana Loes. subsp. harveyana	FoZ #15259
Gymnosporia mossambicensis (Klotzsch) Loes. subsp. mossambicensis	Chase 6279
*Gymnosporia senegalensis* (Lam.) Loes.	FoZ #15183
Hippocratea africana (Willd.) Loes. var. richardiana (Cambess.) N.Robson	Müller 3149
Maytenus acuminata (L.f.) Loes. var. acuminata	Chase 4416
*Maytenus chasei* N.Robson	Chase 5634
*Maytenus undata* (Thunb.) Blakelock	Müller 2803
** Chrysobalanaceae **	
*Parinari curatellifolia* Benth.	FoZ #2039
** Clusiaceae **	
*Garcinia buchananii* Baker	Chase 5296
*Garcinia kingaensis* Engl.	Müller plot 74
*Harungana madagascariensis* Poir.	Chase 1970
Hypericum aethiopicum Thunb. subsp. sonderi (Bredell) N.Robson	FoZ #15321
*Hypericum peplidifolium* A.Rich.	FoZ #15310
*Hypericum revolutum* Vahl	Chase 1181
*Hypericum roeperianum* A.Rich.	Whellan 1138
*Psorospermum febrifugum* Spach	FoZ #15157
** Combretaceae **	
*Combretum molle* G.Don	FoZ #23920
Combretum psidioides Welw. subsp. psidioides	West 6373
** Connaraceae **	
*Agelaea pentagyna* (Lam.) Baill.	Drummond 5079
** Convolvulaceae **	
**Dichondra micrantha* Urb.	FoZ #929
*Hewittia malabarica* (L.) Suresh	FoZ #18123
*Ipomoea cairica (L.) Sweet var. cairica	FoZ #1404
Ipomoea involucrata P.Beauv. var. involucrata	Chase 2163
Ipomoea obscura (L.) Ker Gawl. var. obscura	FoZ #4128
*Turbina holubii* (Baker) A.Meeuse	Chase 8589
** Cornaceae **	
*Curtisia dentata* (Burm.f.) C.A.Sm.	Chase 4168
** Crassulaceae **	
**Bryophyllum pinnatum* (Lam.) Oken	FoZ #25790
**Bryophyllum tubiflorum* Harv.	FoZ #3407
*Crassula alsinoides* (Hook.f.) Engl.	Mavi 1558
*Crassula alticola* R.Fern.	FoZ #39862
Crassula capitella Thunb. subsp. nodulosa (Schönland) Toelken	FoZ #7384
Crassula expansa Dryand. subsp. expansa	FoZ #79967
Crassula lanceolata (Eckl.& Zeyh) Walp. subsp. transvaalensis (Kuntze) Toelken	FoZ #3494
Crassula sarcocaulis Eckl.& Zeyh. subsp. sarcocaulis	Ferrar s.n.
*Crassula sarmentosa* Harv.	FoZ #3499
Crassula setulosa Harv. var. setulosa	Chase 6134
Crassula swaziensis Schönland subsp. swaziensis var. swaziensis	Chase 6149
*Kalanchoe crenata* (Andrews) Haw.	Chase 8445
*Kalanchoe lanceolata* (Forssk.) Pers.	FoZ #1620
Kalanchoe luciae Raym.-Hamet subsp. luciae	FoZ #4061
** Cucurbitaceae **	
*Coccinia adoensis* (A.Rich.) Cogn.	Chase 7388
*Cucumis zeyheri* Sond.	Chase 8485
*Momordica foetida* Schumach.	Wild 14218
*Oreosyce africana* Hook.f.	Chase 6222
*Peponium chirindense* (Baker f.) Cogn.	Müller 3486
*Zehneria minutiflora* (Cogn.) C.Jeffrey	Ferrar 4020
Zehneria scabra (L.f.) Sond. subsp. scabra	Müller plot 169
*Zehneria thwaitesii* (Schweinf.) C.Jeffrey	Chase 5550
** Dipsacaceae **	
*Scabiosa columbaria* L.	FoZ #556
** Dipterocarpaceae **	
*Monotes engleri* Gilg	FoZ #557
** Droseraceae **	
*Drosera burkeana* Planch.	Carly 467
*Drosera dielsiana* Exell & J.R.Laundon	FoZ #644
** Ebenaceae **	
Diospyros abyssinica (Hiern) F.White subsp. abyssinica	Müller 3043
Diospyros lycioides Desf. subsp. sericea (Bernh.) De Winter	Bamps 626
Diospyros natalensis (Harv.) Brenan subsp. nummularia (Brenan) Jordaan	Müller 2556
*Diospyros whyteana* (Hiern) F.White	Chase 1495
** Ericaceae **	
*Erica hexandra* (S.Moore) E.G.H.Oliv.	van der Berghen 623
*Erica johnstoniana* Britten	Galpin 9267
** Erythroxylaceae **	
*Erythroxylum emarginatum* Thonn.	Bamps et al. 648
** Euphorbiaceae **	
*Adenocline acuta* (Thunb.) Baill.	Chase 8491
*Croton sylvaticus* C.Krauss	Drummond 5090
*Euphorbia benthamii* Hiern	Chase 5539
**Euphorbia peplus* L.	FoZ #581
**Euphorbia prostrata* Aiton	FoZ #3742
**Euphorbia tirucalli* L.	Chase 5161
**Homalanthus populifolius* Graham	FoZ #3728
*Leidesia procumbens* (L.) Prain	Wild 6447
*Macaranga capensis* (Baill.) Sim	Chase 5565
*Macaranga mellifera* Prain	Chase 5455
*Neoboutonia macrocalyx* Pax	FoZ #7425
*Ricinus communis L. var. communis	FoZ #25729
*Shirakiopsis elliptica* (Hochst.) Esser (=*Sapium ellipticum* (Krauss) Pax)	Chase 7115
*Suregada procera* (Prain) Croizat	Müller 2557
*Tannodia swynnertonii* (S.Moore) Prain	Müller plot 172
**Vernicia montana* Lour.	FoZ #3799
**Fabaceae: Caesalpinioideae**	
*Bauhinia galpinii* N.E.Br.	FoZ #506
*Bauhinia petersiana* Bolle	FoZ #1030
*Bauhinia variegata L. var. variegata	FoZ #17371
*Brachystegia spiciformis* Benth.	Chase 4153
*Brachystegia utilis* Hutch.& Burtt Davy	Chase 4154
Chamaecrista kirkii (Oliv.) Standl. var. kirkii	Chase 5489
*Chamaecrista parva* (Steyaert) Lock	Ferrar s.n.
*Chamaecrista wittei* (Ghesq.) Lock	Chase 8493
*Julbernardia globiflora* (Benth.) Troupin	FoZ #1040
**Senna didymobotrya* (Fresen.) H.S.Irwin & Barneby	Methuen 43
*Senna petersiana* (Bolle) Lock	FoZ #3923
**Senna septemtrionalis* (Viv.) H.S.Irwin & Barneby	Grosvenor 11
**Fabaceae: Mimosoideae**	
*Acacia abyssinica* Benth.	Chase 7796
*Acacia amythethophylla* A.Rich.	FoZ #15446
*Acacia cornigera* (L.) Willd. (=*A. spadicigera* Schltr. & Cham.)	Obermeyer 2061
**Acacia mearnsii* De Wild.	FoZ #3591
**Acacia melanoxylon* R.Br.	Biegel 2431
**Acacia podalyriifolia* G.Don	FoZ #3800
Acacia sieberiana DC. var. woodii (Burtt Davy) Keay & Brenan	Biegel 2430
*Albizia adianthifolia* (Schumach.) W.Wight	Müller 3383
*Albizia antunesiana* Harms	FoZ #15145
Albizia glaberrima (Schumach.& Thonn.) Benth. var. glabrescens (Oliv.) Brenan	Müller 3097
*Albizia gummifera* (J.F.Gmel.) C.A.Sm.	Wild 1596
Albizia schimperiana Oliv. var. schimperiana	Chase 6223
*Dichrostachys cinerea* (L.) Wight & Arn.	FoZ #15202
*Entada abyssinica* A.Rich.	Chase 5554
*Newtonia buchananii* (Baker f.) G.C.C.Gilbert & Boutique	Chase 878
**Fabaceae: Papilionoideae**	
Aeschynomene nodulosa (Baker) Baker f. var. nodulosa	FoZ #2041
Argyrolobium rupestre (E.Mey.) Walp. subsp. rupestre	Bamps et al. s.n.
*Argyrolobium tomentosum* (Andrews) Druce	FoZ #41255
Craibia brevicaudata (Vatke) Dunn subsp. baptistarum (Büttner) J.B.Gillett	Wild 1586
*Crotalaria capensis* Jacq.	Corby 1582
*Crotalaria cephalotes* A.Rich.	Chase 8565
*Crotalaria chirindae* Baker f.	Hopkins 8033
Crotalaria gazensis Baker f. subsp. gazensis	Müller 6684
*Crotalaria hyssopifolia* Klotzsch	Chase 7055
Crotalaria laburnifolia L. subsp. australis (Baker f.) Polhill	Corby 1445
*Crotalaria lachnophora* A.Rich.	Chase 7449
Crotalaria lanceolata E.Mey. subsp. lanceolata	Chase 7469
Crotalaria pallida Aiton var. pallida	FoZ #5617
*Crotalaria variegata* Baker (=*C. sericifolia* Harms)	Chase 6366
*Dalbergia lactea* Vatke	Müller plot 149
*Dalbergia nitidula* Baker	Chase 168
Desmodium adscendens (Sw.) DC. var. robustum B.G.Schub.	FoZ #35168
Desmodium barbatum (L.) Benth. var. dimorphum (Baker) B.G.Schub.	Staples 183
*Desmodium repandum* (Vahl) DC.	Chase 580
*Desmodium setigerum* (E.Mey.) Harv.	Chase 8557
**Desmodium uncinatum* (Jacq.) DC.	FoZ #3543
Dolichos kilimandscharicus Taub. subsp. kilimandscharicus	FoZ #15323
Dolichos sericeus E.Mey. subsp. sericeus	Chase 6930
Dumasia villosa DC. var. villosa	Whellan 1598
Eriosema buchananii Baker f. var. buchananii	FoZ #52033
*Eriosema burkei* Harv.	Corby 266
Eriosema chrysadenium Taub. var. chrysadenium	FoZ #6264
Eriosema ellipticum Baker subsp. ellipticum	FoZ #1617
*Eriosema psoraleoides* (Lam.) G.Don	FoZ #1697
*Erythrina lysistemon* Hutch.	FoZ #1688
*Flemingia grahamiana* Wight & Arn.	Chase 1739
*Indigofera arrecta* A.Rich.	FoZ #40764
*Indigofera cecilii* N.E.Br.	FoZ #7519
*Indigofera hilaris* Eckl.& Zeyh.	Jacobsen 1488
Indigofera lyallii Baker subsp. lyallii	FoZ #5628
Indigofera paniculata Pers. subsp. gazensis (Baker f.) J.B.Gillett	Chase 6109
Indigofera setiflora Baker var. setifera	Hopkins 7080
Kotschya strigosa (Benth.) Dewit & P.A.Duvign. var. strigosa	Chase 3732
Lablab purpureus (L.) Sw. subsp. uncinatus Verdc. var. uncinatus	Chase 8292
*Lotononis listii* Polhill	FoZ #73221
*Lotus arabicus* L.	Bamps et al. 676
Lotus discolor E.Mey. subsp. mollis J.B.Gillett	Chase 3107
*Lotus namulensis* Brand	Chase 3106
Macrotyloma densiflorum (Baker) Verdc. var. densiflorum	Carter 2128
Mucuna coriacea Baker subsp. irritans (Burtt Davy) Verdc.	FoZ #1699
*Ormocarpum kirkii* S.Moore	Carter 2129
Otholobium foliosum (Oliv.) C.H.Stirt. subsp. gazense (Baker f.) Verdc.	FoZ #2366
*Philenoptera violacea* (Klotzsch) Schrire (=*Lonchocarpus capassa* Rolfe)	Chase 60
Pseudarthria hookeri Wight & Arn. var. hookeri	FoZ #2457
*Pterocarpus angolensis* DC.	FoZ #15141
Pterocarpus rotundifolius (Sond.) Druce subsp. rotundifolius	FoZ #98755
Rhynchosia clivorum S.Moore subsp. pycnantha (Harms) Verdc.	Fischer 1313
*Rhynchosia monophylla* Schltr.	FoZ #3749
*Rhynchosia swynnertonii* Baker f.	Chase 8004
*Sphenostylis zimbabweensis* Mithen	Chase 1530
*Stylosanthes fruticosa* (Retz.) Alston	Staples 185
Tephrosia dasyphylla Baker subsp. dasyphylla	Chase 1964
*Tephrosia festina* Brummitt	Biegel 2432
*Tephrosia meisneri* Hutch.& Burtt Davy (=T. glomeruliflora Meisn. subsp. meisneri (Hutch.& Burtt Davy) Schrire)	Chase 8151
Tephrosia paniculata Baker subsp. paniculata	Chase 7318
Tephrosia rhodesica Baker f. var. polystachyoides (Baker f.) Brummitt	Chase 1966
*Tylosema fassoglensis* (Schweinf.) Torre & Hillc.	FoZ #3927
*Vigna gazensis* Baker f.	Chase 6510
*Vigna schlechteri* Harms	Wild 2844
Vigna vexillata (L.) A.Rich. var. vexillata	FoZ #24719
*Zornia milneana* Mohlenbr.	FoZ #41451
** Gelsemiaceae **	
Mostuea brunonis Didr. var. brunonis	Chase 6745
** Gentianaceae **	
*Sebaea leiostyla* Gilg	Hopkins 7257
** Geraniaceae **	
Geranium arabicum Forssk. subsp. arabicum	FoZ #25127
Geranium incanum Burm.f. subsp. nyassense (R.Knuth) J.R.Laundon	Drummond 5093
*Pelargonium luridum* (Andrews) Sweet	Chase 7921
*Pelargonium mossambicense* Engl.	Eyles 9276
** Gesneriaceae **	
Streptocarpus eylesii S.Moore subsp. eylesii	Plowes 2170
*Streptocarpus hirticapsa* B.L.Burtt	Ferrar s.n.
*Streptocarpus michelmorei* B.L.Burtt	FoZ #627
*Streptocarpus umtaliensis* B.L.Burtt **NE**	Chase 1374
** Halogoraceae **	
**Myriophyllum aquaticum* (Vell.) Verdc.	Denny 1312
** Heteropyxidaceae **	
*Heteropyxis dehniae* Suess.	FoZ #3610
** Hydrostachyaceae **	
*Hydrostachys polymorpha* A.Braun	Chase 6637
** Icacinaceae **	
Apodytes dimidiata Arn. subsp. dimidiata	Chase 366
** Iteaceae **	
*Choristylis rhamnoides* Harv.	Chase 1736
** Lamiaceae **	
*Aeollanthus buchnerianus* Briq.	Fisher 1627
*Aeollanthus serpiculoides* Baker	Whellan 749
Clerodendrum cephalanthum Oliv. subsp. swynnertonii (S.Moore) Verdc.	Chase 2164
*Haumaniastrum dissitifolium* (Baker) A.J.Paton	FoZ #73536
*Haumaniastrum sericeum* (Briq.) A.J.Paton	Hopkins s.n.
*Haumaniastrum villosum* (Benth.) A.J.Paton	Chase 6566
*Hoslundia opposita* Vahl	FoZ #1752
**Hyptis pectinata* (L.) Poit.	FoZ #876
**Hyptis suaveolens* (L.) Poit.	FoZ #27586
Leonotis ocymifolia (Burm.f.) Iwarsson var. raineriana (Vis.) Iwarsson	FoZ #34929
*Leucas milanjiana* Gürke	FoZ #3922
Micromeria imbricata (Forssk.) C.Chr. var. imbricata (=*Satureja punctata* (Benth.) Briq.)	Chase 7137
Ocimum obovatum Benth. subsp. obovatum var. obovatum	FoZ #3364
*Plectranthus chimanimanensis* S.Moore	Chase 6021
*Plectranthus esculentus* N.E.Br.	FoZ #7536
*Plectranthus hadiensis* (Forssk.) Spreng.	FoZ #21128
*Plectranthus hereroensis* Engl.	Chase 6512
*Plectranthus kapatensis* (R.E.Fr.) J.K.Morton	Chase 5563
*Plectranthus lanuginosus* (Benth.) Agnew	FoZ #15345
*Plectranthus laxiflorus* Benth.	Chase 1999
*Plectranthus sanguineus* Britten	FoZ #2052
*Plectranthus swynnertonii* S.Moore	Eyles 7084
*Pycnostachys reticulata* (E.Mey.) Benth.	Chase 2000
*Pycnostachys urticifolia* Hook.	FoZ #37502
*Rotheca myricoides* (Hochst.) D.A.Steane & Mabb.	FoZ #873
**Salvia coccinea* Etl.	FoZ #37444
*Tetradenia riparia* (Hochst.) Codd	Carter 2131
*Vitex doniana* Sweet	Masterson 570
Vitex madiensis Oliv. subsp. milanjiensis (Britten) F.White	Chase 1965
** Lauraceae **	
**Cinnamomum camphora* (L.) J.Presl	FoZ #23896
**Cinnamomum verum* J.Presl	FoZ #25873
*Cryptocarya liebertiana* Engl.	Müller 3382
**Persea americana* Mill.	FoZ #3716
** Lentibulariaceae **	
*Genlisea hispidula* Stapf	Philcox et al. 8951
*Utricularia firmula* Oliv.	Philcox et al. 8952
*Utricularia livida* E.Mey.	Chase 6112
*Utricularia scandens* Benj.	Chase 5539
*Utricularia subulata* L.	Philcox et al. 8950
** Linderniaceae **	
*Craterostigma lanceolatum* (Engl.) Skan	FoZ #53083
Craterostigma sp. no. 1 cf. lanceolatum	FoZ #28553
*Linderniella pulchella* (Skan) Eb.Fisch., Schäferh.& Kai Müll. (=*Lindernia pulchella* (Skan) Philcox)	Philcox 8971
*Linderniella wilmsii* (Engl.) Eb.Fisch., Schäferh.& Kai Müll. (=*Lindernia wilmsii* (Engl.) Philcox)	Philcox 8972
** Loganiaceae **	
*Strychnos angolensis* Gilg	Müller 3801
*Strychnos lucens* Baker	FoZ #1306
*Strychnos spinosa* Lam.	FoZ #15189
*Strychnos usambarensis* Gilg	FoZ #1308
** Loranthaceae **	
*Agelanthus lancifolius* Polhill & Wiens	FoZ #39863
*Agelanthus molleri* (Engl.) Polhill & Wiens	Polhill & Pope 4746
*Agelanthus nyasicus* (Baker & Sprague) Polhill & Wiens	Chase 578
*Englerina oedostemon* (Danser) Polhill & Wiens **NE**	Polhill & Pope 4747
**Malvaceae: Byttnerioideae, Helicteroideae & Sterculioideae**	
Cola greenwayi Brenan var. greenwayi	Drummond 5092
*Dombeya burgessiae* Harv.	Loveridge 1089
*Dombeya rotundifolia* (Hochst.) Planch.	FoZ #1785
*Melhania randii* Baker f.	FoZ #3924
*Waltheria indica* L.	FoZ #37490
**Malvaceae: Grewioideae**	
Grewia occidentalis L. var. occidentalis	FoZ #3190
*Grewia stolzii* Ulbr.	FoZ #7353
*Sparrmannia ricinocarpa* (Eckl.& Zeyh.) Kuntze	Chase 1678
*Triumfetta annua* L.	FoZ #7392
Triumfetta pilosa Roth var. pilosa	FoZ #7393
*Triumfetta rhomboidea* Jacq.	Wild 500
**Malvaceae: Malvoideae**	
*Abutilon sonneratianum* (Cav.) Sweet	Wild 472
*Azanza garckeana* (F.Hoffm.) Exell & Hillc.	FoZ #98753
*Hibiscus fuscus* Garcke	Chase 8301
*Hibiscus shirensis* Sprague & Hutch.	FoZ #7152
*Hibiscus surattensis* L.	Chase 8558
**Malvastrum coromandelianum* (L.) Garcke	FoZ #4040
*Pavonia columella* Cav.	Fisher 1601
*Pavonia urens* Cav.	FoZ #1221
** Melastomataceae **	
*Antherotoma naudinii* Hook.f.	Schelpe 365
Dissotis princeps (Kunth) Triana var. princeps	Whellan 1980
** Meliaceae **	
*Ekebergia benguelensis* C.DC.	FoZ #98848
*Ekebergia capensis* Sparrm.	Chase 555
*Khaya anthotheca* (Welw.) C.DC.	FoZ #25861
*Trichilia dregeana* Sond.	Chase 3506
** Melianthaceae **	
Bersama abyssinica Fresen. subsp. nyassae (Baker f.) F.White	FoZ #98884
*Bersama swynnertonii* Baker f.	Chase 7020
** Menispermaceae **	
*Cissampelos mucronata* A.Rich.	FoZ #37441
*Cissampelos torulosa* Harv.	Chase 1544
Stephania abyssinica (Quart.-Dill.& A.Rich.) Walp. var. abyssinica	Wild 495
Stephania abyssinica (Quart.-Dill.& A.Rich.) Walp. var. tomentella (Oliv.) Diels	FoZ #27674
*Tiliacora funifera* (Miers) Oliv.	Grosvenor 265
** Molluginaceae **	
*Corrigiola drymarioides* Baker f.	Chase 1183
Mollugo cerviana (L.) Ser. var. cerviana	Chase s.n.
** Monimiaceae **	
*Xymalos monospora* (Harv.) Baill.	Chase 6179
** Moraceae **	
Dorstenia buchananii Engl. var. buchananii	Chase 6259
*Ficus chirindensis* C.C.Berg	Müller plot 78
*Ficus craterostoma* Mildbr.& Burret	Plowes 2184
*Ficus exasperata* Vahl	Masterson 602
Ficus natalensis Hochst. subsp. graniticola J.E.Burrows	Hyde s.n.
*Ficus rokko* Warb.& Schweinf. (=*F. thonningii* Blume in part)	Biegel 2490
Ficus scassellatii Pamp. subsp. scassellatii	FoZ #15572
*Ficus sur* Forssk.	FoZ #3525
*Trilepisium madagascariense* DC.	Chase 5625
** Myricaceae **	
*Morella pilulifera* (Rendle) Killick	FoZ #3553
** Myrothamnaceae **	
*Myrothamnus flabellifolius* Welw.	FoZ #2038
** Myrsinaceae **	
*Embelia schimperi* Vatke	Chase 1443
*Maesa lanceolata* Forssk.	Obermeyer 2053
*Myrsine africana* L.	Chase 5295
*Rapanea melanophloeos* (L.) Mez	Chase 6739
** Myrtaceae **	
*Eugenia malangensis* (O.Hoffm.) Nied.	FoZ #1226
*Eugenia natalitia* Sond. (incl. *E. nyassensis* Engl., E. capensis (Eckl.& Zeyh.) Sond. subsp. nyassensis (Engl.) F.White)	Chase 7219
**Psidium cattleianum* Sabine	FoZ #7397
**Psidium guajava* L.	FoZ #3529
*Syzygium cordatum* C.Krauss	Bamps et al. 621
Syzygium guineense (Willd.) DC. subsp. afromontanum F.White (=*S. gerrardii* (Hook.f.) F.White)	Chase 5626
**Syzygium jambos* (L.) Alston	FoZ #25884
** Ochnaceae **	
*Ochna holstii* Engl.	Wild 1594
** Oleaceae **	
*Chionanthus battiscombei* (Hutch.) Stearn	Müller 3077
Chionanthus foveolatus (E.Mey.) Stearn subsp. major (I.Verd.) Stearn	Müller plot 153
*Jasminum abyssinicum* DC.	FoZ #15266
*Jasminum streptopus* E.Mey.	Müller plot 149
Olea capensis L. subsp. macrocarpa (C.H.Wright) I.Verd.	Müller plot 152
*Schrebera alata* (Hochst.) Welw.	FoZ #3586
** Opiliaceae **	
*Opilia amentacea* Roxb.	FoZ #6110
** Orobanchaceae **	
*Alectra sessiliflora* (Vahl) Kuntze (incl. var. monticola (Engl.) Melch. & var. senegalensis (Benth.) Hepper)	Chase 6035, Philcox 8953
*Buchnera speciosa* Skan	Chase 4199
Cycnium adonense Benth. subsp. adonense	Chase 611
*Sopubia ramosa* (Hochst.) Hochst.	Bacon s.n.
*Striga bilabiata* (Thunb.) Kuntze	Wild 2807
*Striga elegans* Benth.	FoZ #24937
** Oxalidaceae **	
*Biophytum umbraculum* Welw.	Chase 5534
**Oxalis corniculata* L.	FoZ #7
**Oxalis latifolia* Kunth	FoZ #5774
Oxalis semiloba Sond. subsp. semiloba	FoZ #15219
** Passifloraceae **	
*Adenia digitata* (Harv.) Engl.	FoZ #37462
Adenia gummifera (Harv.) Harms var. gummifera	FoZ #98866
Adenia lobata (Jacq.) Engl. subsp. rumicifolia (Engl.& Harms) Lye	Müller 3621
*Basananthe apetala* (Baker f.) W.J.de Wilde	FoZ #22042
**Passiflora edulis* Sims	FoZ #3760
** Penaeaceae **	
*Olinia vanguerioides* Baker f.	Fisher 1218
** Peraceae **	
Clutia abyssinica Jaub.& Spach var. abyssinica	Whellan 1599
*Clutia paxii* Pax	FoZ #40916
*Clutia swynnertonii* S.Moore	Galpin 9271
** Phyllanthaceae **	
*Antidesma membranaceum* Müll.Arg.	Müller 3380
*Antidesma venosum* Tul.	FoZ #15191
*Antidesma vogelianum* Müll.Arg.	Hyde s.r.
*Bridelia micrantha* (Hochst.) Baill.	FoZ #3580
Cleistanthus polystachyus Planch. subsp. milleri (Dunkley) Radcl.-Sm. (=*C. apetalus* S.Moore)	Wild 5551
Margaritaria discoidea (Baill.) Webster var. nitida (Pax) Radcl.-Sm.	Chase 5483
*Phyllanthus beillei* Hutch.	Chase 5596
*Phyllanthus ovalifolius* Forssk. (=*P. guineensis* Pax)	Müller plot 79
Uapaca kirkiana Müll.Arg. var. kirkiana	FoZ #596
** Phytolaccaceae **	
*Phytolacca dodecandra* L’Hér.	Müller 3092
**Phytolacca octandra* L.	FoZ #2561
** Piperaceae **	
*Peperomia bangroana* C.DC. (=*P. rotundifolia* sensu FZ)	Müller 3067
Peperomia blanda (Jacq.) Kunth var. leptostachya (Hook.& Arn.) Düll	Chase 851
*Peperomia retusa* (L.f.) A.Dietr.	Wild 2836
*Peperomia tetraphylla* (G.Forst.) Hook.& Arn.	Müller 3061
Piper capense L.f. var. capense	Plowes 2163
** Pittosporaceae **	
Pittosporum viridiflorum Sims var. viridiflorum	Chase 7218
** Plantaginaceae **	
**Linaria vulgaris* Mill.	FoZ #5438
**Plantago major* L.	Ferrar s.n.
**Veronica javanica* Blume	Wild 1591
** Polemoniaceae **	
**Cobaea scandens* Cav.	Edwards s.n.
** Polygalaceae **	
*Polygala gazensis* Baker f.	Schelpe 374
*Polygala ohlendorfiana* Eckl.& Zeyh.	Chase 7873
Polygala virgata Thunb. var. decora (Sond.) Harv.	FoZ #25783
*Polygala wilmsii* Chodat	Obermeyer 2048
*Securidaca longepedunculata* Fresen.	FoZ #3920
** Polygonaceae **	
Oxygonum dregeanum Meisn. subsp. canescens (Sond.) Germish.	Craster s.n.
**Persicaria capitata* (D.Don) H.Gross	FoZ #1294
*Rumex acetosella L. subsp. angiocarpus (Murb.) Murb.	FoZ #2778
**Rumex crispus* L.	FoZ #2781
*Rumex sagittatus* Thunb.	FoZ #2783
** Primulaceae **	
*Ardisiandra wettsteinii* R.Wagner	FoZ #2772
** Proteaceae **	
*Faurea rochetiana* (A.Rich.) Pic.Serm.	Fisher 337
*Faurea rubriflora* Marner	Chase 6045
*Faurea saligna* Harv.	Hyde s.n.
**Grevillea robusta* R.Br.	FoZ #7595
Protea caffra Meisn. subsp. gazensis (Beard) Chisumpa & Brummitt	FoZ #7648
*Protea gaguedi* J.F.Gmel.	FoZ #4872
Protea petiolaris (Hiern) Baker subsp. elegans Chisumpa & Brummitt	Mitchell s.n.
*Protea welwitschii* Engl.	FoZ #1352
** Putranjivaceae **	
Drypetes gerrardii Hutch. var. gerrardii	Hyde s.n.
Drypetes natalensis (Harv.) Hutch. var. natalensis	FoZ #5654
** Ranunculaceae **	
*Clematis brachiata* Thunb.	Müller 3403
*Clematis simensis* Fresen.	Müller 3087
*Ranunculus multifidus* Forssk.	FoZ #98776
*Thalictrum rhynchocarpum* Quart.-Dill.& A.Rich.	FoZ #2887
** Rhamnaceae **	
*Gouania longispicata* Engl.	Simon 925
*Rhamnus prinoides* L’Hér.	Fisher 1334
*Scutia myrtina* (Burm.f.) Kurz	Müller 3391
*Ziziphus mucronata* Willd.	FoZ #5025
** Rhizophoraceae **	
Cassipourea gummiflua Tul. var. verticillata (N.E.Br.) J.Lewis	Chase 1802
*Cassipourea malosana* (Baker) Alston (=*C. congoensis* sensu auct.)	Chase 5454
** Rosaceae **	
*Alchemilla kiwuensis* Engl.	Chase 1162
*Cliffortia serpyllifolia* Cham.& Schltdl.	Chase 1182
**Cotoneaster pannosus* Franch.	FoZ #3811
**Eriobotrya japonica* (Thunb.) Lindl.	FoZ #18306
*Prunus africana* (Hook.f.) Kalkman	Chase 6198
**Prunus cerasoides* D.Don	Bannermann s.n.
**Rubus niveus* Thunb.	FoZ #2697
** Rubiaceae **	
*Afrocanthium ngonii* (Bridson) Lantz (=*Canthium ngonii* Bridson, *C. pseudoverticillatum* sensu R.B. Drumm.)	Müller 3068
Agathisanthemum bojeri Klotzsch subsp. bojeri	FoZ #35444
Aidia micrantha (K.Schum.) F.White var. msonju (K.Krause) Petit	Chase 5552
*Anthospermum ammanioides* S.Moore	FoZ #1592
*Anthospermum herbaceum* L.f.	FoZ #1907
*Anthospermum vallicola* S.Moore	Chase 6037
*Anthospermum whyteanum* Britten	Chase 6038
*Anthospermum zimbabwense* Puff **NE**	FoZ #2034
*Canthium inerme* (L.f.) Kuntze	Chase 5351
*Cephalanthus natalensis* Oliv.	Greenway 8802
**Coffea arabica* L.	FoZ #14119
Coffea mufindiensis Bridson subsp. australis Bridson (=*C. ligustroides* sensu Garcia)	Drummond 5088
*Conostomium natalense* (Hochst.) Bremek.	Chase 6039
*Coptosperma supra*-axillare (Hemsl.) Degreef (=*Tarenna supra*-axillaris (Hemsl.) Bremek. subsp. barbertonensis (Bremek.) Bridson)	Müller 2561
Cremaspora triflora (Thonn.) K.Schum. subsp. triflora	FoZ #99093
*Fadogia ancylantha* Schweinf.	FoZ #786
*Fadogia homblei* De Wild.	FoZ #2219
*Galium chloroionanthum* K.Schum.	Müller plot 82
*Galopina circaeoides* Thunb.	Chase 342
Gardenia imperialis K.Schum. subsp. imperialis	Chase 6555
Heinsenia diervilleoides K.Schum. subsp. diervilleoides	Chase 1466
*Hymenodictyon floribundum* (Hochst.& Steud.) B.L.Rob.	FoZ #3505
*Keetia gueinzii* (Sond.) Bridson (=*Canthium gueinzii* Sond.)	Chase 1810
*Kohautia amatymbica* Eckl.& Zeyh.	FoZ #4405
Leptactina benguelensis (Benth.& Hook.f.) R.D.Good subsp. pubescens Verdc.	FoZ #2042
*Mussaenda arcuata* Poir.	Wild 552
Oldenlandia affinis (Roem.& Schult.) DC. subsp. fugax (Vatke) Verdc.	Chase 2162
Oldenlandia goreensis (DC.) Summerh. var. goreensis	FoZ #3350
Oldenlandia herbacea (L.) Roxb. var. herbacea	Ballings 1734
Otiophora inyangana N.E.Br. subsp. inyangana	Chase 7299
Oxyanthus goetzei K.Schum. subsp. goetzei	FoZ #3772
Oxyanthus speciosus DC. subsp. stenocarpus (K.Schum.) Bridson	Chase 281
Pavetta comostyla S.Moore subsp. comostyla var. inyangensis (Bremek.) Bridson	FoZ #7191
*Pavetta umtalensis* Bremek.	Chase 5490
*Pentanisia schweinfurthii* Hiern	Obermeyer 2029
Pentas purpurea Oliv. subsp. purpurea	Eyles 7081
*Psychotria mahonii* C.H.Wright	Chase 6280
*Psychotria peduncularis* (Salisb.) Steyerm.	Masterson 559b
*Psychotria zombamontana* (Kuntze) E.M.A.Petit	Drummond 5098
*Psydrax kraussioides* (Hiern) Bridson	Müller 25
Psydrax parviflora (Afzel.) Bridson subsp. chapmanii Bridson (=*Canthium vulgare* (K.Schum.) Bullock)	Müller 3050
*Pyrostria bibracteata* (Baker) Cavaco	Müller 3747
**Richardia brasiliensis* Gomes	FoZ #37438
**Richardia scabra* L.	FoZ #3354
Rothmannia fischeri (K.Schum.) Bullock subsp. moramballae (Hiern) Bridson	Chase 6999
*Rothmannia urcelliformis* (Hiern) Robyns	Chase 6210
Rubia cordifolia L. subsp. conotricha (Gand.) Verdc.	Greatrex-SRGH 14938
Rutidea fuscescens Hiern subsp. fuscescens	FoZ #15915
*Rytigynia macrura* Verdc. (=R. sp. 1 of Drummond)	Müller 3595
Sericanthe andongensis (Hiern) Robbr. subsp. engleri (K.Krause) Bridson	FoZ #3509
*Sericanthe* sp. A of FZ	Müller 3082
*Spermacoce natalensis* Hochst.	Hopkins 7096
*Tapiphyllum velutinum* (Hiern) Robyns	Chase 4151
Tarenna pavettoides (Harv.) Sim subsp. affinis (K.Schum.) Bridson	FoZ #3806
Tricalysia coriacea (Benth.) Hiern subsp. angustifolia (J.C.Garcia) Robbr.	Müller plot 68
*Tricalysia pallens* Hiern	Müller plot 91
*Vangueria apiculata* K.Schum.	Müller 2425
*Vangueria esculenta* S.Moore	Müller 3054
Vangueria infausta Burch. subsp. infausta	FoZ #15143
** Rutaceae **	
*Calodendrum capense* (L.f.) Thunb.	Hyde s.r.
**Casimiroa edulis* La Llave	FoZ #21844
**Citrus limon* (L.) Burm.f.	FoZ #4034
Clausena anisata (Willd.) Benth. var. anisata	Whellan 761
*Toddalia asiatica* (L.) Lam.	Bamps et al 625
*Vepris bachmannii* (Engl.) Mziray (=*Oricia bachmannii* (Engl.) I.Verd.)	Chase s.n.
*Vepris nobilis* (Delile) Mziray (=*Teclea nobilis* Delile)	Chase 5297
*Zanthoxylum davyi* (I.Verd.) P.G.Waterman	Müller plot 152
** Salicaceae **	
*Casearia battiscombei* R.E.Fr.	Chase 6177
*Dovyalis lucida* Sim	FoZ #79997
Scolopia stolzii Gilg var. stolzii	Chase 5594
Trimeria grandifolia (Hochst.) Warb. subsp. grandifolia	Drummond 5095
** Santalaceae **	
*Osyridicarpos schimperianus* (A.Rich.) A.DC.	Müller 3594
*Osyris lanceolata* Hochst.& Steud.	FoZ #955
*Thesium ussanguense* Engl.	Bamps et al 691
*Viscum shirense* Sprague	Müller 4744
** Sapindaceae **	
*Alophyllus abyssinicus* (Hochst.) Radlk.	Chase 6226
*Alophyllus africanus* P.Beauv.	Carter 2130
*Alophyllus chirindensis* Baker f.	Müller 3494
Dodonaea viscosa Jacq. subsp. angustifolia (L.f.) J.G.West	Williams 223
*Filicium decipiens* (Wight & Arn.) Thwaites	Chase 1968
*Zanha africana* (Radlk.) Exell	FoZ #15195
*Zanha golungensis* Hiern	Müller 3373
** Sapotaceae **	
*Chrysophyllum gorungosanum* Engl.	Chase 7690
*Englerophytum magalismontanum* (Sond.) T.D.Penn.	FoZ #1383
*Englerophytum natalense* (Sond.) T.D.Penn.	Müller 3622
*Manilkara discolor* (Sond.) J.H.Hemsl.	Chase 4559
*Mimusops zeyheri* Sond.	Chase 1741
** Scrophulariaceae **	
*Buddleja pulchella* N.E.Br.	Müller plot 146
*Buddleja salviifolia* (L.) Lam.	Chase 7798
*Diclis ovata* Benth.	Mavi 1785
*Diclis tenella* Hemsl.	Chase 6119
*Freylinia tropica* S.Moore	FoZ #784
*Hebenstretia angolensis* Rolfe	Chase 6124
Hebenstretia oatesii Rolfe subsp. rhodesiana Roessler	FoZ #24443
*Jamesbrittenia carvalhoi* (Engl.) Hilliard	Chase 259
*Nemesia zimbabwensis* Rendle	Whellan 1559
Selago goetzei Rolfe subsp. ambigua Hilliard	Whellan 1143
** Solanaceae **	
**Cestrum aurantiacum* Lindl.	FoZ #18295
**Nicandra physalodes* (L.) Gaertn.	FoZ #114
**Physalis peruviana* L.	FoZ #1201
**Solanum aculeatissimum* Jacq.	Biegel 2429
*Solanum anguivi* Lam.	Müller 2628
**Solanum betaceum* Cav. (=*Cyphomandra betacea* (Cav.) Sendtn.)	Masterson 1327
*Solanum campylacanthum* A.Rich. (=*S. panduriforme* E.Mey., *S. incanum* auct.)	FoZ #7241
**Solanum lycopersicum* L. (=*Lycopersicon esculentum* Mill.)	FoZ #21088
**Solanum mauritianum* Scop.	FoZ #7344
*Solanum terminale* Forssk.	Drummond 5083
**Sterculiaceae** (see Malvaceae: Byttnerioideae, Helicteroideae & Sterculioideae)	
** Stilbaceae **	
*Halleria lucida* L.	Plowes 2503
*Nuxia congesta* Fresen.	Simon 921
*Nuxia floribunda* Benth.	Plowes 2183
** Thymelaeaceae **	
*Dais cotinifolia* L.	FoZ #4994
Gnidia kraussiana Meisn. var. kraussiana	Chase 7368
*Peddiea africana* Harv.	Chase 1969
*Synaptolepis alternifolia* Oliv.	Chase 6502
**Tiliaceae** (see Malvaceae: Grewioideae)	
** Turneraceae **	
Tricliceras longepedunculatum (Mast.) R.Fern. var. longepedunculatum	Bamps et al 643
** Ulmaceae **	
*Celtis africana* Burm.f.	Chase 7012
*Trema orientalis* (L.) Blume	Bamps et al 728
** Urticaceae **	
*Boehmeria macrophylla* Hornem.	Chase 7135
*Droguetia iners* (Forssk.) Schweinf.	FoZ #3789
*Elatostema monticola* Hook.f.	Müller 803
*Laportea alatipes* Hook.f.	Biegel 3498
*Laportea mooreana* (Hiern) Chew	Chase 6059
Laportea peduncularis (Wedd.) Chew subsp. peduncularis	Müller 3398
*Myrianthus holstii* Engl.	Chase 5790
**Pilea microphylla* (L.) Liebm.	FoZ #37433
*Pilea tetraphylla* (Steud.) Blume	Chase 7487
*Pouzolzia parasitica* (Forssk.) Schweinf.	FoZ #15694
*Urera hypselodendron* (A.Rich.) Wedd.	Chase 8439
*Urera trinervis* (Hochst.) Friis & Immelman	Müller 3498
** Verbenaceae **	
**Lantana camara* L.	FoZ #7593
*Lantana swynnertonii* Moldenke	FoZ #29009
*Lippia javanica* (Burm.f.) Spreng.	FoZ #1552
**Verbena bonariensis* L.	FoZ #1582
**Verbena brasiliensis* Vell.	Bamps et al 617
** Violaceae **	
Rinorea convallarioides (Baker f.) Eyles subsp. convallarioides	Müller 3495
*Rinorea ferruginea* Engl.	Drummond 5081
*Viola abyssinica* Oliv.	Biegel 2423
** Vitaceae **	
*Cayratia gracilis* (Guill.& Perr.) Suess.	Chase 7300
*Cissus petiolata* Hook.f.	Müller 3093
*Cyphostemma buchananii* (Planch.) Wild & R.B.Drumm.	Wild 2834
*Cyphostemma kilimandscharicum* (Gilg) Wild & R.B.Drumm.	Chase 8182
*Cyphostemma montanum* Wild & R.B.Drumm.	FoZ #1776
*Rhoicissus rhomboidea* (Harv.) Planch.	Chase 7394
*Rhoicissus tomentosa* (Lam.) Wild & R.B.Drumm.	FoZ #3574
*Rhoicissus tridentata* (L.f.) Wild & R.B.Drumm.	FoZ #15342
